# Overexpression of *zmm28* increases maize grain yield in the field

**DOI:** 10.1073/pnas.1902593116

**Published:** 2019-11-04

**Authors:** Jingrui Wu, Shai J. Lawit, Ben Weers, Jindong Sun, Nick Mongar, John Van Hemert, Rosana Melo, Xin Meng, Mary Rupe, Joshua Clapp, Kristin Haug Collet, Libby Trecker, Keith Roesler, Layton Peddicord, Jill Thomas, Joanne Hunt, Wengang Zhou, Zhenglin Hou, Matthew Wimmer, Justin Jantes, Hua Mo, Lu Liu, Yiwei Wang, Carl Walker, Olga Danilevskaya, Renee H. Lafitte, Jeffrey R. Schussler, Bo Shen, Jeffrey E. Habben

**Affiliations:** ^a^Research & Development, Corteva Agriscience, Johnston, IA 50131

**Keywords:** MADS-box, *zmm28*, maize yield, carbon assimilation, nitrogen utilization

## Abstract

Increasing maize grain yield has been a major focus of both plant breeding and genetic engineering to meet the global demand for food, feed, and industrial uses. We report that increasing and extending expression of a maize MADS-box transcription factor gene, *zmm28*, under the control of a moderate-constitutive maize promoter, results in maize plants with increased plant growth, photosynthesis capacity, and nitrogen utilization. Molecular and biochemical characterization of *zmm28* transgenic plants demonstrated that their enhanced agronomic traits are associated with elevated plant carbon assimilation, nitrogen utilization, and plant growth. Overall, these positive attributes are associated with a significant increase in grain yield relative to wild-type controls that is consistent across years, environments, and elite germplasm backgrounds.

## Significance

In the approaching decades, food security will likely be more of an issue as there will be an increased demand for grain which will need to be met in an environmentally sustainable manner. To date, commercial transgenic maize has primarily targeted resistance to insects and herbicides. Here we describe a transgenic approach to improve the yield and yield stability of maize. We have demonstrated that increasing and extending the expression of a maize gene, *zmm28*, alters vegetative and reproductive growth parameters and significantly enhances yield in large-scale field trials conducted over multiple years. We conclude that alteration in expression of a native maize gene in maize can create a substantially positive change in a complex trait like grain yield.

More maize grain is produced than any other cereal in the world (https://apps.fas.usda.gov/psdonline/circulars/production.pdf). Maize is a staple food for many populations and is widely used for animal feed and biofuel production and as an industrial raw material. Conventional plant breeding has improved maize productivity and is expected to continue to do so. However, current yield increases are not keeping pace with projected future demand for this cereal (1). A greater understanding of plant genomes and gene function has led to rapid advances in genetic engineering technologies in the last 30 y. As a result of this tremendous effort, many genetic engineering approaches have increased yield in crop plants under laboratory conditions, but the results could not be translated to large-scale field trials. Consequently, only a few publications have shown that genetic engineering can produce transgenic events with improved grain yield above conventional breeding under field conditions (2–7).

MADS-box (named for yeast *Minichromosomal maintenance* [*MCM1*], plant *AGAMOUS* [*AG*] and *DEFICIENS* [*DEF*], and human *Serum Response Factor* [*SRF*]) transcription factors have played a significant role in plant evolution and development (8). MADS-box genes encode homeotic transcription factors which are characterized by a highly conserved DNA-binding domain, called the MADS-box, at the N terminus. They comprise a conserved multigene family in fungi, animals, and plants. MADS domain proteins recognize and bind to specific A/T-rich DNA sequences with a CArG core consensus element to modulate target gene expression (9–11). Most MADS proteins bind either as a homodimer to a CArG-box which has a 10-bp consensus CC[A/T]_6_GG or as a homodimer/heterodimer to a 10-bp consensus CTA[A/T]_4_TAG.

Detailed whole-genome and bioinformatics analyses have identified 142 MADS-box genes in the maize genome (12), about half of which have been functionally characterized (13). Plant MADS-box gene family members play diverse roles in plant growth and development, including modulating genes involved in flowering time regulation, floral organ and meristem development, formation of dehiscence zones, and fruit ripening, as well as embryo, leaf, and root development (14–20). Plant MADS-box genes also play important roles in response to biotic and abiotic stresses (21–23). In addition, some root-expressed MADS-box genes regulate genes involved in lateral root growth and development in response to nitrogen and other nutrient deficiencies (24, 25). Since MADS-box transcription factors can regulate the pathways involved in plant growth and development, they represent an important class of candidates for improving crop yield. However, there has not been a published example of field yield increase in any cereal crop through increased expression of a MADS-box transcription factor.

As part of our transgenic discovery pipeline, hundreds of maize transcription factors (of which a subset were MADS-box genes) were tested, and the MADS-box transcription factor *zmm28* fused to the maize *gos2* promoter, *ZmGos2* (26, 27), provided the most consistent positive grain yield response. This promoter provides moderate constitutive expression relative to the well-characterized maize Ubiquitin promoter (28). In the present study, we show that extending and increasing the expression of the *zmm28* gene can enhance grain yield relative to wild-type (WT) controls. These results were demonstrated in highly replicated field trials conducted across multiple years, locations, and elite germplasm backgrounds. Furthermore, results from multiple physiological, biochemical, and molecular experiments allowed us to propose a model to explain the mechanism of action for enhancing grain yield.

## Results

### *zmm28* Is an AP1-FUL–Like MADS-Box Gene.

**Fig. 1.**
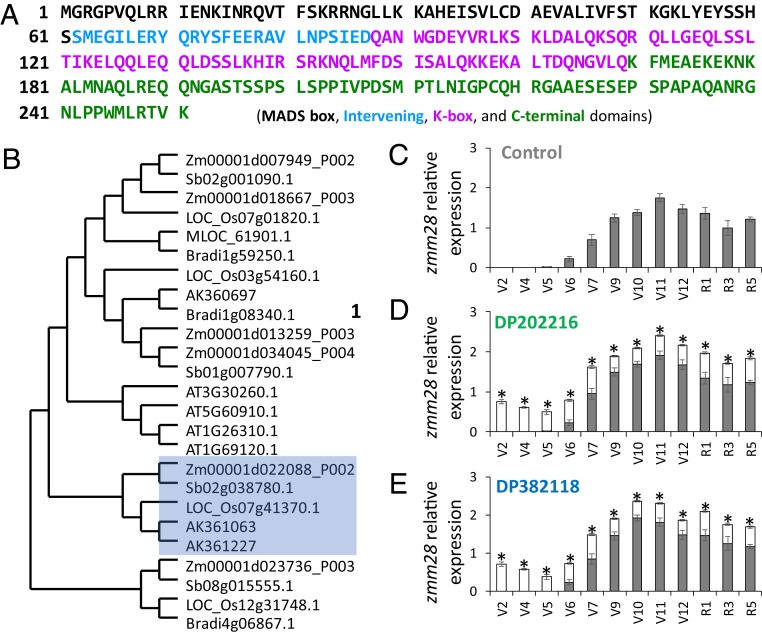
Protein sequence, phylogenetic analysis, and expression of *zmm28*. (*A*) Amino acid sequence of ZMM28. MADS-box, Intervening, K-box, and C-terminal domains are indicated with different colors. (*B*) Phylogenetic analysis of ZMM28 (Zm00001d022088_P002) with AP1-FUL clade members from *Arabidopsis* and representative monocots. The clade containing ZMM28 is highlighted in blue. Relative expression of native (solid bar) and transgenic (open bar) *zmm28* in (*C*) control (WT), (*D*) DP202216, and (*E*) DP382118 leaf tissue during different vegetative (V) stages collected from the middle section of the youngest fully expanded leaf blade and reproductive (R) stages collected from the middle section of the ear leaf blade. Total (native and transgenic) *zmm28* expression is significantly greater in transgenic events than in the WT at all growth stages (*n* = 5 for V2 to R1, *n* = 4 for R3 and R5; *P < 0.05, ±SEM).

The *zmm28* gene (UniProtKB–Q84V67) encodes a MIKC-type MADS-box transcriptional factor where the MADS (M) domain is followed by Intervening (I), Keratin-like (K), and C-terminal domains (29) (Fig. 1*A*) and belongs to the AP1-FUL clade of MADS-box genes. Phylogenetic analysis of ZMM28 (Zm00001d022088_P002; synonym MADS67) with the AP1-FUL clade MADS-box genes from *Arabidopsis*, rice, sorghum, barley, Brachypodium, and maize is shown in Fig. 1*B*. ZMM28 clusters with and shares 94, 75, 69, and 76% amino acid sequence identity with sorghum Sb02g038780.1, barley AK361063 and AK361227, and rice LOC_Os07g41370.1 (OsMADS18) proteins, respectively. The MADS-box genes in this clade control plant development, such as the transition from vegetative to reproductive growth, as well as determination of floral meristem and floral organ identity (30–32).

### Native *zmm28* Expression in WT Maize.

RNA analysis in WT maize demonstrated that native *zmm28* transcripts can be detected in leaf, root, stem, shoot apical meristem (SAM), tassel, and ear (Fig. 1*C* and *SI Appendix*, Fig. S1). However, native *zmm28* transcript and ZMM28 protein are undetectable until V6 (6 fully expanded leaves) in leaves and peak at V11 (Fig. 1*C* and *SI Appendix*, Fig. S1*C*). Overall, the greatest transcript expression and protein levels in leaves occur during ear and tassel formation (from V9 to V12 stages), and they remain relatively high in both vegetative and reproductive tissues.

### Transgenic Event Selection.

An expression cassette was created that contained the *zmm28* coding sequence controlled by the *ZmGos2* promoter (26) coupled with the maize *Ubi1* intron 1 and a *pinII* terminator (hereafter referred to as *ZmGos2-zmm28*; *SI Appendix*, Fig. S2*A*). The *ZmGos2*:*Ubi1* intron 1 combination gives moderate constitutive gene expression in maize (28, 33). Transformation of semielite inbred maize (PH17AW) with *ZmGos2-zmm28* resulted in 52 single-copy, Agrobacterium-backbone–free, *zmm28* overexpression events. Field tests of the 52 events demonstrated that 46 of them had greater grain yield than WT controls. Six of the highest yielding events were selected for expanded field testing to confirm their yield efficacy. Across multitesting locations, the 6 transgenic events showed a yield increase of 302.4, 269.9, 253.3, 224.6, 214.6, and 186.2 kg ha^−1^, relative to the WT (*P* < 0.05). Molecular characterization showed that 2 of the transgenic events, DP-202216-6 and DP-382118-7 (hereafter DP202216 and DP382118, respectively), contained all genetic elements from the T-DNA. In addition, T-DNA junction analysis with Southern-by-Sequencing (SbS) (34) demonstrated only a single junction for each border sequence on the T-DNA in each event showing insertion at a single, nonrepetitive, noncoding locus in the genome (*SI Appendix*, Fig. S2 *B* and *C*). These 2 transgenic events were used for introgression of the transgene expression cassette into elite inbred lines, PHR1J and PHW2Z, for advanced grain yield testing in elite hybrids and transgene mechanism of action studies.

### Native and Transgene *zmm28* Expression in Transgenic Maize.

Compared to *zmm28* expression in WT plants, expression of *zmm28* in the 2 transgenic events was observed in earlier growth stages (V2 to V5) in the leaf due to the presence of the transgenic *zmm28* and then had a similar expression profile to that of WT from V6 through senescence (Fig. 1 *D* and *E*). Expression of *zmm28* is also found in root, stem, SAM, tassel, and ear in the 2 events (*SI Appendix*, Fig. S1 *A* and *B*). Concentrations of ZMM28 protein in the leaf in the events followed a similar temporal profile to that for mRNA levels and were detected at sub-parts per million levels (*SI Appendix*, Fig. S1*C*). However, there was no relationship between transcript level and protein expression in the root (*SI Appendix*, Fig. S1*D*).

Subcellular localization experiments of *ZmGos2-zmm28* transgenic protein with either an N-terminal or C-terminal *Aequorea coerulescens* green fluorescent protein (AcGFP1) tag indicated localization to the nucleus in independent stably transformed maize events (*SI Appendix*, Fig. S2*D*). This result was confirmed in transient assays including a nuclear marker protein, histone H2B (Zm00001d032070), fused to mKate2 (*SI Appendix*, Fig. S2*E*).

### Transgenic *zmm*28 Events Have Increased Grain Yield Relative to WT Controls.

An extensive series of large-scale field trials was conducted from 2014 to 2017 to test the capability of the 2 selected *zmm28* transgenic events to increase grain yield in elite hybrid germplasm relative to WT controls. Over this 4-y period, a total of 48 hybrids ranging in relative maturity from 105 to 113 d were evaluated at 58 locations (*SI Appendix*, Table S1).

**Fig. 2.**
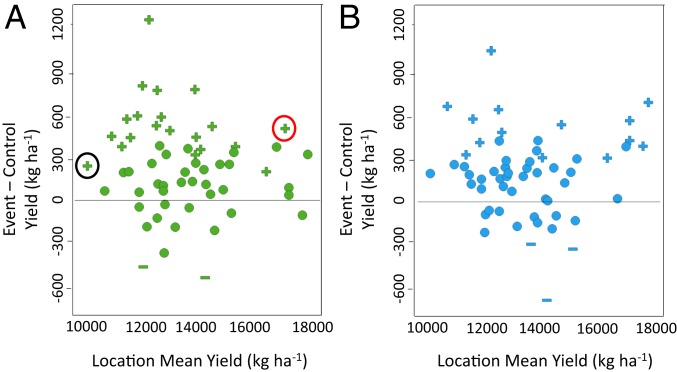
The effect of *ZmGos2-zmm28* on grain yield measured in multiyear, multilocation field experiments. (*A*) DP202216 and (*B*) DP382118 yield differences from WT controls. Each data point represents the difference in yield of the event to the WT control (*y* axis) across 6 to 12 hybrids relative to the overall yield level of the location (x axis). Plus and minus indicate significantly greater or lower than the control (*P* < 0.05), respectively, and dot represents no significant difference of the event relative to the control (*P* < 0.05). The black encircled symbol indicates a significantly positive yield increase of 12 DP202216 hybrids relative to the controls at a low-yielding location. The red encircled symbol indicates a significantly positive yield increase of the same 12 DP202216 hybrids relative to the controls at a high-yielding location.

Statistical analysis of the grain yield of DP202216 and DP382118 showed a strong positive response of the transgenic events relative to their WT controls when averaged across all hybrids within a location (Fig. 2 *A* and *B*). Interestingly, the positive yield response in both events can be observed across a majority of the environments ranging from ∼10,230 to 18,200 kg ha^−1^. For example, in 1 y, there were 12 DP202216/WT hybrid combinations that were grown at a lower-yielding location (symbol encircled in black; Fig. 2*A*), and the transgenic hybrids outyielded the WT controls by 300 kg ha^−1^. When this same set of hybrids was tested at a higher-yielding location in the same year (symbol encircled in red; Fig. 2*A*), the 12 hybrids expressing the transgene still outperformed the controls on average by 400 kg ha^−1^. Overall, across the 58 locations, DP202216 and DP382118 outyielded the WT controls 79 and 78% of the time, respectively.

### Extended and Increased *zmm28* Expression Results in Maize Plants with Altered Agronomic Traits.

**Fig. 3.**
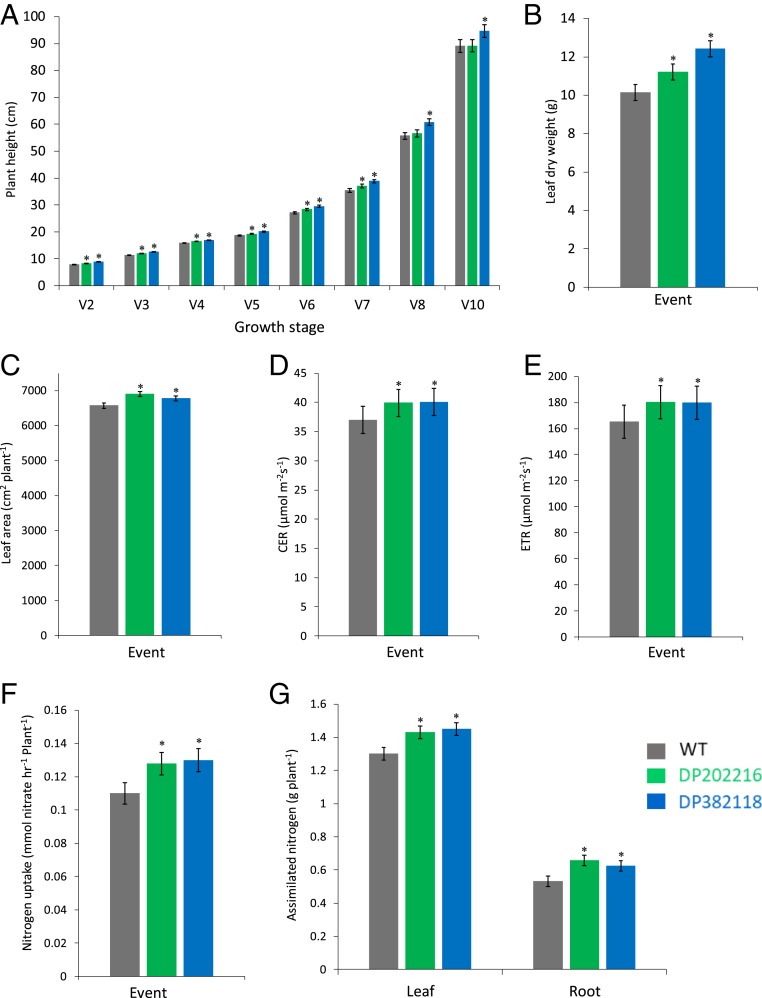
Physiological characterization of WT, DP202216, and DP382118 maize plants. (*A*) Seedling plant height from V2 to V10 growth stages in greenhouse-grown plants, *n* = 96 for V2, V3, V5, V7, and V8; *n* = 144 for V4 and V6; and *n* = 48 for V10 plants. (*B*) Dry weight of leaves at the V8 stage in greenhouse-grown plants, *n* = 48. (*C*) Total leaf area at R1 stage in field pot-grown plants, *n* = 30. (*D*) Leaf photosynthetic CER at V11 stage in field pot-grown plants, *n* = 60. (*E*) Photosynthetic ETR at V11 in field pot-grown plants, *n* = 60. (*F*) N uptake in plants grown in hydroponic conditions, *n* = 31 for WT and *n* = 29 for both DP202216 and DP382118. (*G*) Assimilated N content in leaf and root in field pot-grown plants, *n* = 19 for WT and DP382118 and *n* = 20 for DP202216. *P ≤ 0.05, ±SEM for all figures.

A series of morphometric measurements was performed to identify plant growth parameters in the *ZmGos2-zmm28* events that were altered relative to WT. The extended and increased *zmm28* gene expression resulted in plants with greater vegetative stage plant height, leaf biomass, and total leaf area (Fig. 3 *A*–*C*). Plant height of both DP202216 and DP382118 was significantly greater than that of WT controls from V2 to V7, averaged across all tested hybrids (Fig. 3*A*). Leaf dry weight (Fig. 3*B*) was 11 and 22% greater for DP202216 and DP382118, respectively, at the V8 stage. Furthermore, both *ZmGos2-zmm28* events had an average of 4% greater R1 leaf area, compared to the WT controls (Fig. 3*C*).

We also measured typical plant breeding secondary traits. As shown in *SI Appendix*, Table S2, percent differences between transgenic events and WT were significant for final plant height, heat units to tassel shed, heat units to ear silking, and grain moisture; however, these differences were small in magnitude (0.3 to 1.5%). Importantly, correlations between grain yield and plant height, as well as between grain yield and grain moisture, were not significant (for event DP202216, R^2^ values are 0.003 and 0.038 for yield relative to plant height and grain moisture, respectively; for event DP382118, R^2^ values are 0.016 and 0.001 for yield relative to plant height and grain moisture, respectively). Thus, these secondary traits are unlikely to have a demonstrable impact on enhancing grain yield in the transgenic plants.

### Extended and Increased *zmm28* Expression Increases Leaf Photosynthesis.

Photosynthesis is an important factor in determining crop yield, and thus, we ascertained whether this attribute was altered in the transgenic events. Photosynthesis, expressed as CO_2_ exchange rate (CER) and photosynthetic electron transport rate (ETR), was measured under photosynthetic photon flux density (PPFD) 1,800 µmol m^−2^ s^−1^ from field pot-grown DP202216 and DP382118 plants in 1 inbred and 3 hybrid backgrounds, together with their WT controls, at the V11 growth stage. As shown in Fig. 3 *D* and *E* across the 4 germplasm backgrounds, the CER and ETR were on average increased by 8 and 9%, respectively, in both DP202216 and DP382118. Furthermore, leaf photosynthesis of the *zmm28* transgenic events in response to different light levels was compared to WT with the same set of plants at R4 growth stage. The results demonstrated that overexpression of *zmm28* increased photosynthesis in both events across multiple light levels, and the differences are more pronounced at saturated PPFDs (*SI Appendix*, Fig. S3).

### Extended and Increased *zmm28* Expression Increases N Uptake and Assimilation.

Improving nitrogen (N) utilization is an often-sought mechanism to increase crop yield (35). We first investigated whether extended and increased expression of *zmm28* could improve N uptake. DP202216, DP382118, and WT plants were grown hydroponically in a growth chamber and were analyzed at the V8 stage. Our results demonstrated that N uptake was significantly greater by 16% in DP202216 and 18% in DP382118 compared to WT controls (Fig. 3*F*). In addition, nitrogen assimilation, measured as the total N content minus NO_3_^−^ in leaf and root tissues, was significantly greater in DP202216 and DP382118. At the R1 stage, DP202216 and DP382118 had increased assimilated N over that of WT controls: 10 and 12%, respectively, in the leaf and 23 and 17%, respectively, in the root (Fig. 3*G*).

### Extended and Increased *zmm28* Expression Improves C4 Photosynthetic Enzyme Activities and Nitrate Reductase Activity, but Not Glutamine Synthase Activity.

Gas exchange studies (Fig. 3*D*) revealed increases in CO_2_ fixation rate in both DP202216 and DP382118. To determine whether photosynthesis-relevant enzyme activities were altered in the events, specific activities of major C4 photosynthetic enzymes were examined at 2 growth stages, V4 and V11 (Table 1). These 2 growth stages were chosen because at V4, only transgenic *zmm28* is expressed, while at V11 both native and transgenic *zmm28* are transcribed. Statistically significant increases in activities were observed in 1 or both transgenic events for PEPC and NADP-MDH at V4 and for PEPC, NADP-MDH, and PPDK at V11 (Table 1). The most consistent effect was observed for NADP-MDH, with both events at V11 and event DP382118 at V4 having a significant increase in enzyme specific activity. These increases in the C4 photosynthetic enzyme activities are in good agreement with the increase in CO_*2*_ fixation rate.

**Table 1.** Effects of *ZmGos2-zmm28* on specific activities of selected maize leaf C4 photosynthetic enzymes measured in samples of V4 and V11 stages maize hybrids grown in greenhouse conditions

Harvest stage	Statistical parameter	WT	DP202216	DP382118
PEPC (µmol mg^−1^ min^−1^)				
V4	Mean estimate ± SE	0.445 ± 0.0104	0.464 ± 0.0104	0.494 ± 0.0104
	*P* value	–	0.2232	0.0058**
V11	Mean estimate ± SE	0.668 ± 0.0194	0.724 ± 0.0194	0.718 ± 0.0194
	*P* value	–	0.0633*	0.0950*
NADP-MDH (µmol mg^−1^ min^−1^)				
V4	Mean estimate ± SE	0.434 ± 0.0227	0.477 ± 0.0227	0.514 ± 0.0227
	*P* value	–	0.2097	0.0293**
V11	Mean estimate ± SE	0.661 ± 0.0291	0.762 ± 0.0291	0.772 ± 0.0291
	*P* value	–	0.0309**	0.0195**
NADP-ME (µmol mg^−1^ min^−1^)				
V4	Mean estimate ± SE	0.324 ± 0.0142	0.354 ± 0.0142	0.359 ± 0.0142
	*P* value	–	0.1627	0.1144
V11	Mean estimate ± SE	0.347 ± 0.0117	0.331 ± 0.0117	0.322 ± 0.0117
	*P* value	–	0.3785	0.1708
PPDK (µmol mg^−1^ min^−1^)				
V4	Mean estimate ± SE	0.0435 ± 0.00160	0.0459 ± 0.00160	0.0468 ± 0.00160
	*P* value	–	0.3085	0.1694
V11	Mean estimate ± SE	0.0350 ± 0.00130	0.0397 ± 0.00130	0.0374 ± 0.00130
	*P* value	–	0.0240**	0.2171

*n* = 6, *P < 0.10, **P < 0.05, ±SEM.

Nitrate reductase (NR) catalyzes the rate-limiting step in nitrate assimilation by initiating reduction of nitrate to organic forms, and this enzyme is well established as a key regulator of N assimilation and acquisition (36). Thus, NR specific activity was assayed in leaf and root tissues from DP202216, DP382118, and WT plants at the V4 and V11 growth stages. The results showed that NR enzyme activity was significantly increased in DP202216 and DP382118 in leaf tissue at both growth stages (Table 2). The increase of NR activity is consistent with the N uptake and assimilation results in Fig. 3 *F* and *G*. However, there was no significant difference in NR activity in root tissue between the 2 events and WT control lines.

**Table 2.** Effects of *ZmGos2-zmm28* on specific activities of maize leaf nitrate reductase measured in samples of V4 and V11 stages maize hybrids grown in greenhouse conditions

Harvest stage	Statistical parameter	WT	DP202216	DP382118
Nitrate reductase (nmol mg^−1^ min^−1^)				
V4	Mean estimate ± SE	3.37 ± 0.200	3.97 ± 0.236	4.62 ± 0.275
	*P* value	–	0.0596*	0.0006**
V11	Mean estimate ± SE	2.41 ± 0.114	2.95 ± 0.139	3.85 ± 0.182
	*P* value	–	0.0100**	<0.0001**

*n* = 6, *P < 0.10, **P < 0.05, ±SEM.

The specific activity of another key N assimilation enzyme—glutamine synthetase (GS)—was also examined in the same tissue samples used for the NR assays. GS activities did not show a significant difference between DP202216 and DP382118 and WT controls in either leaf or root. This may indicate that no significant additional GS activity is needed for the increased N assimilation measured in DP202216 and DP382118.

### Protein–Protein Interactions with ZMM28.

**Fig. 4.**
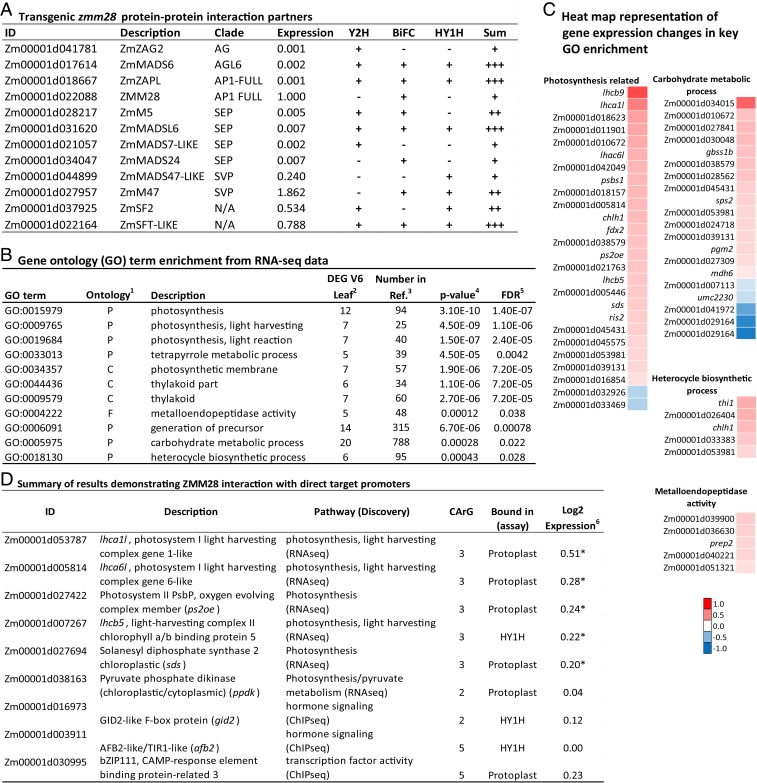
Data summary of transgenic ZMM28 protein–protein interactions, transcription modulation, and analyzed direct targets. (*A*) Summary of protein–protein interactions with potential contribution to transgenically expressed *zmm28* mechanism of action. For context, DP202216 V6 maize leaf RNA-seq protein interactor expression values are normalized to *zmm28* expression. (*B*) GO term enrichment of DP202216 V6 leaf tissue transcriptomic analysis. (*C*) Pathway analysis of differentially expressed gene transcripts in event DP202216 V6 leaf tissue. The heat map displays Log_2_ fold change of differentially expressed genes functioning in GO term enriched pathways. GO terms 0015979, 0009765, 0019684, 0006091, 0033013, 0034357, 0044436, and 0009579 are grouped as “photosynthesis related.” (*D*) Summary of promoter direct target analysis and expression in V6 leaves of event DP202216. BiFC, maize protoplast BiFC; HY1H, heterodimer yeast 1-hybrid. Superscript numbers are as follows: 1, P is biological process, F is molecular function, and C is cellular component; 2, number of genes associated with each GO term that are differentially expressed between WT and event DP202216 V6 leaf; 3, total number of genes in each GO category expressed in V6 leaf total detected transcripts; 4, Fisher’s exact test for GO term enrichment; 5, false discovery rates; and 6, V6 leaf. Asterisk indicates statistically significant (adjusted *P* < 0.05).

MADS-box transcription factors associate as homodimers or heterodimers to bind CArG box elements and subsequently modulate target gene expression. To identify protein–protein interaction partners that potentially interact with native ZMM28 protein, yeast 2-hybrid (Y2H) screening was performed with a B73 immature ear library resulting in the identification of 6 potential MADS-box protein–protein interaction partners (Fig. 4*A*).

Since native *zmm28* does not express at early growth stages, protein–protein interaction partners contributing to the transgenic maize events phenotypes in seedlings and young leaves were assayed using Y2H screening of a PH184C seedling (V2 to V3) library and a B73 V3 to V7 leaf library. Nine total interacting proteins, none of which are MADS-box proteins, were identified from the 2 libraries. Interactions with 2 of these proteins, ZmSF2 and ZmSFT-like, were supported by further experiments as shown in Fig. 4*A*.

Potential interaction partners of ZMM28 were further tested in vivo with a bimolecular fluorescence complementation (BiFC) assay (37). Following transfection of maize protoplasts, fluorescence was measured indicating interaction between nGFP-Prey and cGFP-ZMM28 (Bait) (*SI Appendix*, Table S3). As BiFC is prone to false-positive self-assembly independent of protein–protein interaction (38), flow cytometry was used to quantify the BiFC signal and reduce the occurrence of false positives (39). All signal comparisons were made to a negative control providing a baseline for self-assembly. The control was created by deleting 47 amino acids from the leucine zipper-like K-domain of ZMMADSL6, a known protein interaction partner of ZMM28 identified from bioinformatics prediction and Y2H experiment. Truncated ZMMADSL6 (ZMMADSL6-MUT) had significantly reduced interaction with ZMM28 relative to WT ZMMADSL6 while still maintaining nuclear localization. Of the 12 tested protein interactions, 8 were experimentally supported via the BiFC assay with almost half the interactions being positive in both BiFC and Y2H assays (Fig. 4*A*).

### Transcriptome Analysis of Maize Event DP2022216.

Transcriptome analysis was carried out to identify differentially expressed genes (DEGs) and their associated pathways that could provide a possible molecular basis for the previously described increased photosynthesis, N utilization, and plant growth. For simplicity, RNA-seq analysis was conducted on V6 leaves from DP202216 and WT plants. Results of this analysis identified 192 up-regulated and 64 down-regulated transcripts in DP202216 leaves as compared to the WT leaf data (*SI Appendix*, Table S4). CArG box sequences were contained within 3 kb upstream of their promoters in 76% of the DEGs, relative to 26 to 28% of DEGs from 2 overexpressed non-MADS transcription factors, ZmWUS and ZmNAC7, over a total of 4 experiments. These results suggest that many of the DEGs may be directly regulated by transgenic ZMM28 binding to their promoters at the V6 stage.

To further gain an overarching view of the DEG function, Gene Ontology (GO) enrichment analysis was conducted, and 11 GO terms were identified in the V6 leaf DEG dataset (Fig. 4 *B* and *C*). Photosynthesis, generation of precursor metabolites and energy, and carbohydrate metabolic processes were the 3 main GO terms identified, all of which could contribute to promote plant growth and development. These results are consistent with the measured phenotypes of *ZmGos2-zmm28* plants and suggest that expression of photosynthesis and carbon assimilation-related genes could be associated with positive vegetative and reproductive phenotypes measured in the transgenic events.

### Identification of Direct Targets of Transgenic ZMM28.

To identify genes directly modulated by transgenic ZMM28 and their associated pathways, genomic sequences directly bound by ZMM28 were recovered from leaves of WT and DP202216 plants at the V4 stage (at which time there was no detectable native ZMM28 protein) and analyzed by chromatin immunoprecipitation and sequencing (ChIP-Seq). In addition, putative direct targets of the transgenic ZMM28 were identified from CArG-motif enrichment of the promoters from the strong differentially regulated DEGs in the transcriptome experiment.

Two in-cell assays were used to collectively validate candidate direct target promoters identified from the above 2 experiments. A heterodimer yeast 1-hybrid (HY1H) assay analyzed the capability of ZMM28 and 1 of its protein–protein interaction partners as listed in Fig. 4*A* to directly bind a promoter. Additionally, V2 etiolated maize protoplast cells were used in a protoplast direct-target assay using a ZsGreen1 reporter to detect ZMM28 interactions with promoters. The HY1H assay provided predetermined heterodimer interaction partners while the maize protoplast direct-target assay potentially tested ZMM28 homodimers or heterodimers, forming between native protein–protein interaction partners. Promoters of key photosynthetic pathway components were bound by ZMM28, as were promoters of gibberellin and auxin receptor genes which are responsible for sensing these phytohormones (Fig. 4*D*).

## Discussion

MADS-box transcription factors have been shown to regulate genes involved in controlling numerous plant growth and development characteristics (14). In particular, a group of MADS-box genes, including *zmm28*, have been targets of selection during maize domestication, indicating that they play vital roles in regulating genes involved in plant architecture and selected traits such as grain yield (40). Here we demonstrated, using more than 2,400 replications of field data, that alteration of expression of a native maize gene, *zmm28*, via genetic engineering can significantly enhance the grain yield of maize relative to WT controls. These data were collected in commercially relevant hybrids over multiple years in testing locations with a wide range in average overall yield levels. In addition, our molecular, biochemical, and physiological characterizations provide insights into possible mechanisms contributing to the positive attributes measured in the *zmm28* transgenic events.

Given that *zmm28* has been a target of selection during maize domestication, we infer that its variation has been essentially exhausted during this advancement. However, by using transgenic technology to extend and increase *zmm28* expression, we created relevant diversity which resulted in greater maize yield. Importantly, this improvement in plant performance occurred with no undesirable secondary phenotypes, which typically occur in plants with transgenically overexpressed transcription factors (41).

From a spatial standpoint, the expression analysis showed that the native *zmm28* gene is expressed in leaf, root, stem, stalk, SAM, tassel, ear, and kernel tissues in WT plants. From a temporal perspective, native *zmm28* transcript and protein were only detected in maize after the V6 stage. The greatest levels of both *zmm28* transcript and protein in maize leaf coincide with the development and formation of ears and tassels. A close homolog of *zmm28* in rice, *OsMADS18*, functions in regulating genes involved in the vegetative-to-reproductive phase change and genes that promote flowering (42). The protein sequence conservation and similarity of *zmm28* and *OsMADS18* expression (42) suggest that they may share some overlapping functions in their respective crops. Indeed, in both DP202216 and DP382118 events, there is a statistically significant shift in silking and shedding. However, these changes are negligible in magnitude (<1%) leading us to surmise that altered expression of zmm28 in maize is distinct from that of OsMADS18 in rice.

We demonstrated that modulation of *zmm28* expression improves multiple vegetative phenotypes. These include an increase of early plant vigor, measured as an increase in plant height and leaf biomass, as well as an increase of total leaf area. These changes collectively could contribute to a greater source capacity in both carbon assimilation and nitrogen utilization as well as a strengthened sink at the whole-plant level. Recently, Trachsel et al. (43) demonstrated that early seedling vigor is positively correlated with final grain yield in multiple conventional maize hybrids across numerous environments. Maize yield has increased 4-fold since 1950, a result explained in part by increases in source activity per unit land area, combined with an increase in effective filling period (extended stay-green) (44). These increases in resource capture required concomitant increases in sink size to accommodate the utilization of those resources for greater yield. This balance between source and sink activity can be modulated by the growing environment. For example, as plant density increases, source availability per plant decreases, creating a more source limited environment (45). As plant population density has increased over the last 50 y in North American maize production (46), the need for improved resource capture at the plant level is evident to support both vegetative and reproductive sinks.

Photosynthesis provides essential assimilates for biomass accumulation as well as for reproductive growth and development. Conventional breeding has achieved significant improvements in canopy photosynthesis by altering leaf angle and selecting for delayed senescence (47). However, neither traditional breeding nor genetic engineering approaches have been successful in directly improving leaf photosynthetic efficiency to increase crop yield (48). Interestingly, increased and extended expression of *zmm28* in maize not only results in greater leaf area at both vegetative and reproductive stages, which could increase light interception and source capacity at the canopy level, but also increases photosynthesis rate per unit leaf area. This is supported by observed increases in CER, ETR, and enzyme activity of key C4 cycle photosynthetic enzymes, as well as increased transcription of photosynthesis-related genes. The increased photosynthesis rate and greater leaf area could contribute to measured plant biomass and grain yield enhancement.

A net increase in photosynthetic capacity must be balanced with utilization of the elevated resources by an adequately sized sink capacity (49). The increased sink strength from the expanding leaves and potential for storage of hemicelluloses in stems at vegetative stages could sustain the higher carbon assimilation in the transgenic plants (50), whereas up-regulated photosynthetic capacity above the utilization capacity of developing reproductive sinks (ears) can lead to excessive biomass accumulation in vegetative tissues (45). There are practical limits to the value of vegetative growth in commercial maize breeding programs. Often, selection for larger plants is capped to prevent an excessive increase in plant height, which can introduce greater susceptibility to agronomic issues such as lodging. As indicated in *SI Appendix*, Table S2, the slight increase in plant height observed in *zmm28* plants did not increase root lodging, suggesting the maintenance of acceptable agronomics in the transgenic events is coincident with greater biomass accumulation and associated partitioning of assimilates into vegetative and reproductive sink tissues.

**Fig. 5.**
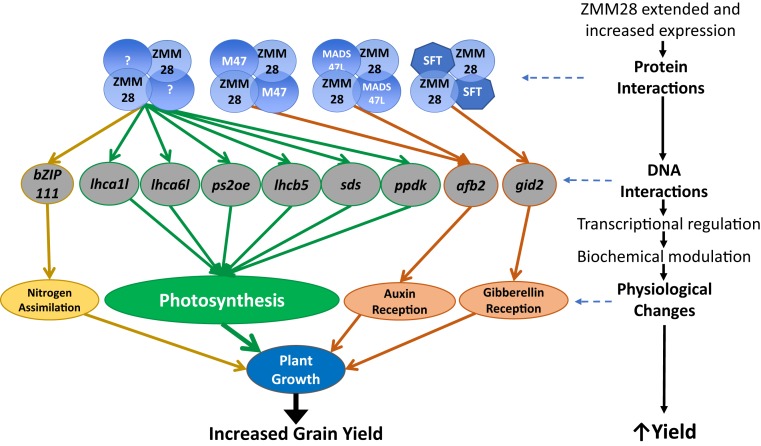
Model representing proposed mechanism of action for *ZmGos2-zmm28* in maize. Distinct ZMM28 protein complexes (in blue) (Fig. 4*A*) interact with direct target gene promoters (in gray) (Fig. 4*B*) to regulate transcription, with downstream impacts on biochemical and physiological processes including photosynthesis (in green), nitrogen assimilation (in yellow), and growth regulating hormone reception (in coral) leading to enhanced plant growth and increased grain yield. Question mark represents unidentified transgenic ZMM28 protein–protein interaction partners.

Photosynthesis and nitrogen metabolism are closely linked by their interdependence for fixed carbon, chemical energy, and nitrogen assimilates. The maintenance of an ideal C and N balance within plants is critical for optimal plant growth and development. Typically, greater photosynthesis and increased growth occur in parallel with an increased demand for nitrogen utilization or vice versa (51, 52). Our results showed that *ZmGos2-zmm28* plants can enhance nitrogen metabolism by increasing the nitrogen uptake rate and assimilation capacity. Coupling this with the increase in photosynthesis as well as auxin and gibberellin signaling, *ZmGos2-zmm28* plants have enhanced vegetative and reproductive performance (Fig. 5). Moreover, the enhanced N utilization of these transgenic events suggests that they could serve as a starting point to create more environmentally sustainable maize hybrids, with increased grain yield.

Transcript profiling of DP202216 showed a small number of differentially expressed genes changing at a modest magnitude compared to that of many other plants with modified transcription factor expression (53–55). Despite this, transcript profiling suggests that photosynthesis light and dark reactions are enhanced in DP202216. Chlorophyll binding proteins, chlorophyll biosynthetic precursor pathways (tetrapyrrole biosynthesis), and carbohydrate biosynthetic process steady state transcript levels were up-regulated in V6 transgenic leaves. This associates with phenotypic and biochemical evidence of up-regulated photosynthesis in this tissue.

Many of the identified ZMM28 protein interaction partners are members of the MADS-box SEP and AG clades, which are known to interact with one another in the formation of protein quartets and play an important role in floral development (56). Protein–protein interactors with expression early in development are most relevant to transgenic ZMM28 function. The interaction with broadly expressed MADS-box proteins ZmM47 and ZmMADS47-like provide a likely mechanism of interaction with promoters in the transgenic events, as does the potential for ZMM28 homodimerization.

Direct DNA targets bound by the early (V2 to V6) expressed ZMM28 included an auxin-receptor AFB2 ortholog, a gibberellin-receptor GID2 ortholog, and 4 genes encoding proteins in photosynthetic light-harvesting pathways. These direct targets of transgenic ZMM28 imply modulation of key hormone sensing pathways (auxin and gibberellin) as well as stimulus of light-harvesting reactions of photosynthesis. In a separate study, we overexpressed an *Arabidopsis* ortholog of the direct target *ZmbZIP111*, *AtbZIP9*, and found it increased both *Arabidopsis* greenness and rosette area at 13 d of growth under low nitrogen conditions (0.4 mM KNO_3_), at *P* values of 2.17 e^−5^ and 0.058, respectively. The regulation of *ZmbZIP111* by the transgenic ZMM28 suggests a pathway for carbon/nitrogen balance regulation.

Transcription factors are important targets for improving complex traits in crop plants, since they can function as master regulators of many cellular processes that regulate growth, development, stress tolerance, and grain yield (57). However, the fundamental challenge with manipulating transcription factors for crop improvement is to express them in the right tissue, at the right developmental stage, and at the ideal expression level to avoid negative pleiotropic effects. We tested multiple promoters, including a set of constitutive and tissue specific promoters, coupled with different introns to optimize *zmm28* expression for grain yield improvement. Among the 13 promoter/intron combinations tested, the moderate constitutive promoter *ZmGos2* combined with the maize *Ubi1* intron 1 controlling *zmm28* expression showed the most consistent grain yield efficacy without negative pleiotropy.

In a recent GWAS study, *zmm28* was listed as a candidate transcription factor for controlling flowering traits (58). When compared to parents of the NAM population, *zmm28* had a wide range in transcript abundance among the 14 NAM founders (59). However, it is difficult to pinpoint how much of that variation is due to genetics versus differences in developmental timing. To further assess the native function of *zmm28*, null mutants have been created and will be analyzed.

Enhancement of carbon and nitrogen resource capture by source tissues, coupled with efficient resource utilization by sink tissues, is required to drive improvements in grain yield of maize. By using a transgenic approach, we have favorably altered key agronomic traits, such as plant height, leaf biomass, photosynthesis rate, and nitrogen utilization. These attributes are difficult to directly alter through conventional breeding due to their complex nature and quantitative inheritance. In addition, altered expression of *zmm28* created stronger sinks as evidenced by the increase in vegetative growth and grain yield. Based on our physiological, biochemical, and molecular characterization, we postulate that the molecular action of the *ZmGos2-zmm28* construct creates enhanced leaf source capacity, increased N uptake and assimilation, and increased growth resulting in enhanced performance in elite hybrids (Fig. 5). Overall, we conclude that alteration in expression of a single native gene in maize, *zmm28*, can improve both resource capture and resource utilization, resulting in a significant improvement in grain yield, the ultimate complex quantitative trait.

## Materials and Methods

Complete details concerning plant materials, experimental methods, experimental designs, and data analyses are provided in *SI Appendix*.

## Supplementary Material

Supplementary File
