# Supplementary material for: Overexpression of zmm28 increases maize grain yield in the field
Source: Proc Natl Acad Sci U S A. 2019 Nov 4;116(47):23850–8. doi: 10.1073/pnas.1902593116 (PMC6876154; doi:10.1073/pnas.1902593116)
Supplement: Supplementary File [file pnas.1902593116.sapp.pdf]

PNAS
www.pnas.org

Supplementary Information for

Overexpression of *zmm28* Increases Maize Grain Yield in the Field

Jingrui Wu, Shai J. Lawit, Ben Weers, Jindong Sun, Nick Mongar, John Van Hemert, Rosana Melo, Xin Meng, Mary Rupe, Joshua Clapp, Kristin Haug Collet, Libby Trecker, Keith Roesler, Layton Peddicord, Jill Thomas, Joanne Hunt, Wengang Zhou, Zhenglin Hou, Matthew Wimmer, Justin Jantes, Hua Mo, Lu Liu, Yiwei Wang, Carl Walker, Olga Danilevskaya, H. Renee Lafitte, Jeffrey R. Schussler, Bo Shen, Jeffrey E. Habben

Corresponding authors: Jingrui Wu, Email: jingrui.wu@corteva.com
Jeffrey E. Habben, Email: jeffrey.habben@corteva.com

This PDF file includes:

Materials and Methods
Figs. S1 to S4
Tables S1 to S4
Reference for the Materials and Methods

www.pnas.org/cgi/doi/10.1073/pnas.1902593116

Materials and Methods

Generation and molecular characterization of maize transgenic events and elite conversions of *ZmGos2-zmm28* inbreds

Transgenic maize plants were generated with an *Agrobacterium tumefaciens*-based transformation cassette *ZmGos2-zmm28* (*SI Appendix,* Fig. S2*A*). Expression of *zmm28* is controlled by the promoter from the *Zea mays* translation initiation factor *gos2* (*zm-gos2*) gene, ZmGos2 (1) operationally fused to the intron 1 region from the maize *ubiquitin 1* (*zm-ubi1*) gene (2), conferring moderate constitutive-expression. Transcription of the *zmm28* gene cassette is ended by the terminator sequence from the *proteinase inhibitor II* (*pinII*) gene from *Solanum tuberosum* (3). Transformation was into a semi-elite inbred maize line (PH17AW) and resulted in numerous single copy, backbone free *zmm28* overexpression (*ZmGos2-zmm28*) events. The *ZmGos2-zmm28* PH17AW served as the donor for standard backcrossing of two lead events, DP202216 and DP382118. Event integrity, copy number and insertion site were analyzed using the next generation sequencing method, SbS (4). Introgression of both events was made into two elite inbred lines, PHR1J and PHW2Z, one each from the two major heterotic groups, stiff stalk and non-stiff stalk, respectively. Hybrids were then created and tested with the two elite inbreds (PHR1J and PHW2Z) of DP202216 and DP382118.

Maize hybrid yield testing and statistical analysis

To evaluate the effect of *ZmGos2-zmm28* on grain yield, field trials were conducted in North and South America from 2014-2017. Across the four-year period, a total of 48 hybrids were evaluated with DP202216 and DP382118 events. For each hybrid, a *ZmGos2-zmm28* homozygous inbred conversion line (for each transgenic event) was top-crossed to a select inbred tester to generate F1 seed. For the WT control hybrid, a non *ZmGos2-zmm28* transformed inbred line was top-crossed to the corresponding select inbred tester to generate F1 seed. All subsequent comparisons were made between the heterozygous F1 transgenic hybrid and F1 WT control of the same hybrid. During the four years period, experimental entries were evaluated across a range of environments at testing sites located in in Woodland, CA; Plainview, TX; Garden City, KS; York, NE; Union City, TN; Johnston, IA; Adel, IA; Marion, IA; Readlyn, IA; Reasnor, IA; Miami, MO; Sikeston, MO; Sciota, IL; San Jose, IL; Buda, IL; Princeton, IL; Humboldt, IL; Seymour, IL; Windfall, IN; Volga, SD; Janesville, WI; Mankato, MN; and Viluco and Buin, Chile. All testing sites were established and managed with the goal of achieving optimal yield levels. Fifty-eight testing sites provided data of sufficient quality across the four-year period. Average yield levels ranged from 10,230 kg ha^{-1} to 18,200 kg ha^{-1} across those sites.

Experimental designs were split plots with hybrid background as the main plot and event or the WT as the sub-plot. Two to three replicates were established at each testing site with main plots randomized within replication and sub-plots randomized within main plot. Experimental entries were grown in four-row plots that ranged from 4.4 m to 5.3 m in length with a 0.5 m alley in between. Using a standardized quality control procedure, outlier data points were identified and eliminated before final statistical analysis. Grain weights and moistures for each experimental entry were measured by harvesting the center two rows of each four-row plot using a small-plot research combine. Yield was standardized within the experiment by adjusting the harvested grain weight of each plot to fifteen percent moisture. Analysis was conducted using ASREML (VSN International Ltd), and the values are presented as BLUEs (Best Linear Unbiased Estimates) (5-7).

In the analysis for grain yield, main effect of event is considered as fixed effects and hybrid background and interaction between event and hybrid background are treated as random effects. The blocking factors such as replicates, its interaction with hybrid background and field spatial variation such as row and column effects are considered as random. To analyze yield, autoregressive correlation as AR1*AR1 was included. The significance test between both

DP202216 and DP382118 against the WT within and across hybrids was performed using a p-value of 0.05 in a two-tailed test. The multiple comparisons correction was not applied.

Plant growth conditions

For field-plot experiments, plants were grown in Johnston, Iowa. Experimental design was a split plot as described above, with hybrids as main plots and transgenic events and WT as sub-plots, 4 - 12 replications, and 4-row plots (4.4 m row length). The field was managed per standard agronomic practice for annual corn/soybean rotation with normal fertilizer application and had neither drought stress nor nitrogen deficiency.

For field-pot experiments, plants were grown in 10 L pots (Treepot TP818, Stuewe & Sons, Inc). The pots were filled with either a custom soil mixed in-house with peat, bark, perlite and vermiculite or a mixture of 50% vermiculite (Hummert International) and 50% Turface™ MVP (Profile Products, LLC). The pots were placed in wooden support racks (L 250 cm x W 60 cm x H 55 cm) that held 12 x 2 pots with plants potted approximately 19 cm apart in each rack and were arranged in the field (Johnston, IA). Each row contained 6 racks with a 60 cm space between each row. Buffer plants were planted around each experiment and consisted of 1 row on each side and 4 x 2 pots at the end of each row. Total 10 rows including the two border rows for buffer plants were planted in the experiment. Plant spacing approximated a planting density of 84210 plants ha^{-1}, and was comparable to plant populations for maize production in the Corn Belt. Experimental designs were set up as a randomized complete block or as a split-plot with hybrids as the main plot and transgenic or WT as the sub-plot. The plants were watered with 100 ppm Peters® Excel 15-5-15 fertilizer (Everris NA Inc) to maintain well-watered and normal nitrogen conditions.

For greenhouse experiments, plants were grown in 6 L pots with a mix of 50% Turface™ MVP (Hummert International) and 50% vermiculite (Hummert International). The experiments were nested by harvest time, then blocked by hybrids, and the transgenic events and WT controls were randomized in each hybrid block. The plants were given supplemental lighting, a 16-h light and 8-h dark photoperiod, temperature of 26 °C day/20 °C night and relative humidity of 60%. The plants were watered with 100 ppm Peters® Excel 15-5-15 fertilizer to maintain well-watered and normal nitrogen conditions.

For hydroponic nitrogen uptake experiments, the plants were grown hydroponically in growth chambers with a photoperiod of 16-h light and 8-h dark, photosynthetic photon flux density (PPFD) of 400 - 600 $\mu mol\ m^{-2}\ s^{-1}$ under metal halide lights, temperatures of 25 °C day/20 °C night, and relative humidity of 70%. Seeds were germinated for six days in 25 cm x 46 cm germination paper rolls. Each seedling was inserted into a 5 cm-long foam plug cut from slit insulation pipe (Cat# 3W-299, Grainger, Lake Forest, IL) and inserted into a 1 L amber rectangular bottle (Cat# 414004-199, VWR, Radnor, PA). The plants were grown in modified Hoagland solution that was aerated continuously and replaced once a day. The modified hydroponic solution consisted of 1 mM $MgSO_4$, 0.5 mM KH_2PO_4, 1 mM $CaCl_2$, 80 ppm Sprint Fe330 [Sequestrene 330; Ciba-Geigy, Greensboro, NC], 3µM H_3BO_3, 2 µM $MnCl_2$, 2 µM $ZnSO_4$, 1 µM $CuSO_4$, 0.12 µM $NaMoO_4$, and 4 mM nitrate nitrogen (8, 9).

Plant secondary traits collection from field plots

Plant height was recorded by taking the average height of four plants per plot and was measured from the base of plant to the flag leaf node. Heat Units to Silk and Shed were recorded as the total accumulated heat units from planting until 50% of the plants within a plot had exerted silks or anthers, respectively. Root Lodging percentage was captured as the percent of plants within a plot that had root lodging angles that resulted in stalk to ground angles of less than 45 degrees. Grain moisture was determined at the time of harvest using grain moisture probes inserted in the weigh buckets of small plot research combines. Grain moisture and total grain weight of each plot are used in combination to calculate yield per plot standardized to a constant grain moisture of 15%. Each measurement was taken at the individual plot level for a total of "n" measurements or plots

per trait across the four-year period. A statistical significance test between each event and the wild type was performed using a p-value of 0.05 in a two-tailed test.

Plants used for physiological, biochemical and molecular analyses

One inbred and one to five different hybrids of *ZmGOS2-zmm28* were used in physiological, biochemical and molecular studies based on maturity, grain yield, seed availability and the size of the experiments. In the greenhouse early seedling assays, four hybrids were tested, including PH12SG x PHW2Z, PH17RD x PHW2Z, PHR1J x PH1W4R and PHR1J x PHVAM. In the total leaf area test, five hybrids were measured from field-plots, including PHR1J x PH26V11, PHR1J x PH45J21, PHR1J x PHCPW, PHR1J x PH1W4RBM1 and PHR1J x PHVAMBM1. CO_2 exchange and electron transport rates were measured on one inbred, PH17AW and three hybrids, PH17AW x PH1HJC1, PHR1J x PHVAM and PHW2Z x PH12PC in a field-pot experiment. Nitrogen uptake assays were performed with two hybrids PHR1J x PHVAM and PH17RD x PHW2Z in growth chambers. Nitrogen assimilation was tested with field-pot grown PHR1J x PHVAM. The activities of C4 photosynthetic enzymes and nitrate reductase and GS were assayed with PHR1J x PHVAM and PH12SG x PHW2Z. The native gene and transgene expression as well as protein levels were tested with PHR1J x PHCPW from field-plots and PHR1J x PHVAM from the greenhouse. The RNA-seq and ChIP-seq experiments were conducted with PH17AW x PH1HJC1.

Protoplasts were isolated from non-transgenic inbred R03 for BiFC and ZMM28 direct target assays. For transgenic ZMM28 subcellular localization, HI-II x Gaspe Flint was used for stable expression of *ZmGOS2-zmm28*-AcGFP1.

Sample collection for *zmm28* gene expression and ZMM28 protein concentration determinations

Each leaf sample was obtained by cutting the youngest fully-expanded leaf blade at vegetative stages and ear leaf blade at reproductive stages. The tissue was cut into sections of 2.5 cm^2 and collected into vials. Each stalk sample was obtained by cutting a section of the internode below the fully expanded leaf at vegetative stages and below the ear leaf at reproductive stages. Each shoot apical meristem (SAM) sample was obtained by dissecting shoot apices with one to two leaf primordia attached from six seedlings. Each root sample was obtained by digging a circle 25-38 cm in diameter around the base of the plant to a depth of 18-23 cm. The roots were thoroughly cleaned with water and a representative sample was removed from the plant. No above ground brace roots were included in the sample. The root tissue was cut into sections 2.5 cm or smaller in length and collected into vials. Each pollen sample was obtained by bagging tassels and shaking or tapping the bag to dislodge pollen. The pollen was screened for anthers and foreign material, and then collected into vials. Silk samples were collected from shoot-bagged ears (silks not exposed to pollen). After removing the shoot bag, exposed silks were cut and placed into sample vials. Each kernel sample was obtained from self-pollinated ears. For each sample, a representative sub sample of 15 kernels was collected into a vial.

Each sample was frozen immediately with liquid nitrogen in the field or greenhouse and was transferred to the laboratory in dry ice. For gene expression analysis, the samples were ground with a mortar and pestle in liquid nitrogen, after which the powdered sample was immediately aliquoted into 1.2 mL pre-chilled bullet tubes and stored frozen until analysis. For protein concentration determination, the samples were lyophilized under vacuum until dry. Following lyophilization, leaf, root, stalk and kernel samples were finely homogenized and stored frozen until analysis.

***zmm28* native gene and transgene expression determination**

For expression analysis, total RNA was extracted using Qiagen RNeasy reagents with synthesis of cDNA done with Applied Biosystems High Capacity cDNA Reverse Transcription kits.

Quantitative PCR was done with hydrolysis probe and SYBR based reactions. Primers and probes were designed using Applied Biosystems Primer Express software using nucleotide sequences published in Genbank. Hydrolysis probe-based PCR was performed using Bioline Sensi-fast mix while SYBR-based PCR was run using Applied Biosystems PowerUp SYBR Green Master Mix. All reactions were run on the Applied Biosystem Viia7 Real-Time PCR instrument using manufacturer's conditions. Relative gene expression was calculated by normalizing against *eukaryotic initiation factor 4-gamma* (Zm00001d004676).

ZMM28 protein concentration determinations

For leaf, root, and stalk, the concentration of ZMM28 protein was determined using a quantitative enzyme linked immunosorbent assay (ELISA). Samples were extracted with 0.60 mL of chilled buffer comprised of 0.25% ASB-14 in phosphate buffered saline containing polysorbate 20 (PBST). Extracted samples were centrifuged, and then supernatants were used for analysis. Standards and samples were incubated in a plate pre-coated with a ZMM28-specific monoclonal antibody (8H10). Following incubation, and washing, a second ZMM28-specific polyclonal antibody (R743), conjugated to the enzyme horseradish peroxidase (HRP), was added to the plate and incubated. Following washing, detection of the bound ZMM28-antibody complex was accomplished by the addition of substrate, which generated a colored product in the presence of HRP. The reaction was stopped with an acid solution and the optical density (OD) of each well was determined using a plate reader. A standard curve was included on each ELISA plate. The equation for the standard curve used a quadratic fit to relate the OD values obtained for each set of standard wells to the respective standard concentration (ng mL^{-1}).

For kernel tissue, the concentration of ZMM28 protein was determined using a semi-quantitative Western blot assay. A standard curve was prepared in extracted grain matrix. Samples were extracted with 0.60 mL of lithium dodecyl sulfate with dithiothreitol sample reducing buffer. Extracted samples were centrifuged, and the supernatants removed and prepared for analysis. Treated standards and samples were heated at 95 °C for 5 min, dispensed to a 4-12% Bis-Tris gel, and separated using Polyacrylamide gel electrophoresis (PAGE). Proteins were transferred to a nitrocellulose membrane using the iBlot dry blotting apparatus (Thermo Fisher Scientific, USA). Following transfer, the membrane was blocked and incubated in a 1:3000 dilution of a mouse monoclonal antibody (8H10) specific to the ZMM28 protein, washed, and then incubated in a secondary anti-mouse antibody, which was conjugated to the enzyme horseradish peroxidase (HRP) and diluted 1:5000. Chemiluminescent substrate was added to the membrane for signal detection and the Western blot image was captured using an imaging system. The densitometry values obtained were related to the respective standard concentration (ng mL^{-1}).

Adjusted sample concentration values were converted from ng mL^{-1} to ng mg^{-1} sample weight as follows:

$$\text{Sample Concentration (ng protein mg}^{-1}\text{ sample weight)} = \text{Sample Concentration (ng mL}^{-1}\text{)} \times \frac{\text{Extraction Buffer Volume (mL)}}{\text{Sample Target Weight (mg)}}$$

Individual sample results below the limit of detection (LOD) were assigned a value of zero. Results between the LOD and the assay lower limit of quantification (LLOQ) were assigned a value equal to half the LLOQ for calculation purposes.

***ZmGos2-zmm28* transgenic protein subcellular localization**

To determine the subcellular localization of the transgenic ZMM28 protein, both N-terminal and C-terminal GFP fusion proteins were produced using the monomeric AcGFP1 (Clontech, USA/Takara, Japan) driven by *ZmGos2* promoter/*zm-ubi1 intron 1*. Stable transformation was performed as above. A maize histone H2B fusion with mKate2 (10) (Evrogen, Russia) was

expressed from an Arabidopsis *ubiquitin 10* promoter as a nuclear marker. Particle bombardment into B73 seedlings was performed as in Lawit *et al.* (11). The etiolated seedlings were observed after 23 h recovery at room temperature. The AcGFP1-positive coleoptile cells were visualized with a Leica (Wetzlar, Germany) DMRXA epifluorescence microscope as in Lawit *et al*. (12). The mKate2 fluorescence was observed using a filter set from Chroma Technology (Bellows Falls, VT): Cy5 #41008 (exc. 590-65, dichroic 660LP, em. 663-737).

Plant height (V2 – V10), leaf dry weight and total leaf area measurements

Plant height was measured from the soil surface to the collar of the youngest fully expanded leaf. For leaf dry weight measurements, all the leaves were collected from each individual plant, placed in a paper bag and dried at 70 °C for 72 h, or until there was no further weight change with time. The samples were weighed after equilibrating at room temperature for 1 h. Total leaf area was measured using field-plot grown plants. Briefly, the plants were collected from the field-plot by cutting the stalk at the soil surface at the R1 growth stage, then all the leaves from an individual plant were excised and leaf area of each leaf was measured with a Li-3100C leaf area meter (Li-Cor, Lincoln, NE USA).

Nitrate determination

Nitrate concentration was determined based on Miranda *et al*. (13). Nitrate was reduced to nitrite by vanadium, and nitrite was detected by the acidic Griess reaction using spectrophotometric absorbance. A 200 µL nitrate sample was mixed with 50 µL of saturated vanadium solution (VCl_3 in 1 M HCl) and 50 µL Griess solution (0.04% N-(1-Naphthyl) ethylenediamine dihydrochloride and 2% sulphanilamide in 5% HCl, made fresh daily) in each well of a 96-well plate, covered and incubated at 37 °C for 3.5 h. The absorbance at 540 nm was read with a plate reader (Synergy HTX, BioTek, Winooski, VT). Nitrate content was calculated based on standard nitrate solutions. All chemicals were from Sigma-Aldrich (St. Louis, MO).

Total nitrogen quantification

Total tissue nitrogen analysis was performed by combustion analysis on a Flash 1112EA analyzer (Thermo) configured for N/Protein determination as described by the instrument manufacturer. 25-35 mg subsamples were weighed and recorded to an accuracy of 0.001mg on a Mettler-Toledo MX5 microbalance and were prepared in tin capsules prior to analysis. Percent N values were determined using the Eager 300 software based on a calibration (K factor) calculation using known standards.

Nitrogen uptake and assimilation

Nitrogen uptake was estimated based on nitrate depletion from a hydroponic solution at the V8 growth stage. One day before harvest, nitrate was added to each of the bottles at a final concentration of 8 mM. Liquid samples were collected from each of the bottles at the beginning for an estimate of the initial nitrate concentration and after 24 h for an estimate of the final nitrate concentration.

Nitrogen assimilation was estimated based on the reduced nitrogen in the plant. Shoot biomass and root biomass were collected separately at harvest and the total tissue nitrogen and nitrate levels were determined. Nitrogen assimilation was estimated based on the difference between the total combustion nitrogen and nitrate in the plants.

Gas exchange and chlorophyll fluorescence

Gas exchange and chlorophyll fluorescence were measured using portable gas exchange systems (LI-6400 LCF; LI-COR, Lincoln, NE) as described previously (14). The CO_2 sensors and water vapor sensors of the gas exchange systems were calibrated using gas of a known CO_2 level with 21% oxygen and nitrogen as balance, and known water vapor concentrations generated with a

controlled humidification system (LI-610 Portable Dew Point Generator; LI-COR, Lincoln, NE). The temperature, relative humidity, photosynthetic photon flux density (PPFD) and CO_2 levels were set at 26 °C, 70%, 1800 µmol m^{-2} s^{-1} and 400 ppm, respectively, in leaf chambers. PPFD levels were controlled using a chamber integrated red-blue light source with 10% blue light for all the measurements. Data were collected on the youngest fully expanded leaves at V11 on field-pot grown plants on sunny days. Each plant was measured five times by auto-logging in the data in 5 min measurements, and the average value of the five measurements is considered as one replicate for each entry. ETR was calculated as ($\Phi_{PSII} \times PPFD \times \alpha \times \beta$), where Φ_{PSII}, the quantum yield of non-cyclic electron transport was calculated as $(F'_m - Fs)/F'_m$. The light absorptivity coefficient, α, was assumed to be 0.87, and the factor for the partitioning of photons between incident PSII and PSI, β, was assumed to be 0.40 (15). Photosynthesis light response curve (AQ) generated at PPFD 2000, 1800, 1500, 500, 100 and 0 µmol m^{-2} s^{-1} , 3 min at each light level, with ear leaves at R4 under the same conditions as described above.

Sampling for enzyme assays

Leaf and root samples from maize plants grown in a greenhouse were harvested at growth stages V4 and V11, frozen in liquid nitrogen, ground by hand under liquid nitrogen with a mortar and pestle, and stored at -80°C. Two plants were combined for each sample. At V4, the entire 3rd and 4th leaves were sampled. At V11, the middle sections of the 11th leaf were sampled. To allow for the expression of enzyme activity on a per mg protein basis, protein concentrations of all extracts were determined by the Bradford method using the Coomassie Plus reagent (Thermo Scientific) with BSA as standard.

Photosynthetic enzyme activity assays

The C4 photosynthetic enzyme assays were adapted from published methods of Kanai and Edwards (16) and Krall *et al*. (17). The assays were performed at 25 °C in 96-well plates with a final volume of 200 µL using the change in absorbance at 340 nm and a molar extinction coefficient of 6.22 x 10^3 M^{-1} cm^{-1} for NADPH and NADH. Typically, 30 to 50 mg of ground leaf powder was extracted with 6 µL buffer per mg of leaf powder, at 4 °C for 15 min with an inverting mixer. Centrifugation at 20,000 x g was done twice (1 min, then 5 min) and the 2nd supernatant was kept on ice. The extraction buffer for NADP-ME and NADP-MDH assays was 50 mM Tris-HCl pH 8, 5 mM $MgCl_2$, and 25 mM DTT. The assay buffer for NADP-ME was 50 mM Tris-HCl pH 8, 2.5 mM EDTA, 5 mM DTT, 0.4mM $NADP^+$, 5 mM L-malate, and 20 mM $MgCl_2$. Sample was added and the background rate (usually negligible) was monitored for several minutes, prior to starting the reaction with $MgCl_2$. NADP-MDH assays were performed following a 2-h incubation of the extracts on ice to ensure full reductive activation by DTT. The assay buffer for NADP-MDH was 50 mM Tris-HCl pH 8, 5 mM DTT, 0.2 mM NADPH, and 3 mM OAA. Sample was added, background was monitored for several minutes until stable, and then the reaction was started with OAA. The extraction buffer for PEPC and PPDK was 50 mM Tris-HCl pH 8, 20% glycerol, 10 mM $MgCl_2$, and 5 mM DTT. The assay buffer for PEPC was 50 mM Tris-HCl pH 8, 20% glycerol, 10 mM $MgCl_2$, 10 mM $NaHCO_3$, 0.2 mM NADH, 3 U of MDH, and 4 mM PEP. Sample was added, and the plate was incubated at 25 °C for 20 min before starting the reaction with PEP. For PPDK, the assay buffer was 50 mM Tris-HCl pH 8, 20% glycerol, 5 mM DTT, 10 mM $MgCl_2$, 5 mM $(NH_4)_2SO_4$, 2.5 mM KH_2PO_4, 10 mM $NaHCO_3$, 2 mM pyruvate, 0.2 mM NADH, 3 U MDH, and 1.25 mM ATP; the reaction was started with ATP. Additional PEPC was not added because PEPC activity in the extract was in great excess compared with PPDK activity. PPDK activity increased with time, so the assays were done for 45 min to get a stable linear decrease in A_{340nm}.

Nitrate reductase assay

The assay was performed in PCR tubes at 30 °C for 30 min in a volume of 100 µL using an endpoint spectrophotometric assay. Extractions were performed with 45 - 55 mg of leaf tissue with 5 µL

buffer per mg of leaf powder, at 4 °C for 10 min. Centrifugation was performed twice for 5 min at 12,000 x g and once for 1 min at 20,000 x g; the 2nd supernatant was kept on ice. The extraction buffer was 50 mM HEPES, pH 7.5, 5 mM EDTA, 1:100 Halt Protease and Phosphatase Inhibitor, 1% (w/v) PVPP, and 2 mM DTT. The assay buffer was 50 mM HEPES pH 7.5, 1 mM NaF, 10 mM KNO3, 0.17 mM NADH, and 5 mM EDTA. Sample was added, and the reaction was performed for 30 min, then stopped with 100 µL of stopping solution. Stopping solution was 1% sulfanilamide in 1.5 N HCl and 0.02% N-(1-naphthyl)-ethylenediaminehydrochloride. Color development occurred for 10 min at RT prior to measuring absorbance of the samples at A_{540nm}.

Yeast two-hybrid assay

The Matchmaker Gold Yeast Two-Hybrid System (Clontech, USA/Takara, Japan) was used to discover and test for potential protein interaction partners with ZMM28. Three maize cDNA prey libraries were constructed from B73 V12 - V14 immature ear, PH184C V2 - V3 whole seedling, and B73 V3 - V7 leaf RNA. The cDNA libraries were generated using SMART technology and co-transformed with linearized pGADT7-Rec into Yeast Strain Y187. At least one million prey clones from each library were mated to a ZMM28 bait strain. Mating was continued until zygotes could be observed using a light microscope and then plated on QDO/-Ade/-His/-Leu/-Trp and incubated at 30° C for 5 days. Identified protein interaction partners were re-transformed into the Y2H system for confirmation testing.

Bimolecular fluorescence complementation (BiFC)

Coding sequences for candidate protein-protein interaction partners to ZMM28 were synthesized by GenScript (USA) and placed under the control of the *ZmGos2* promoter with a *ZmUbi* intron 1. The coding sequences were translationally fused to the C-terminal or N-terminal part of the monomeric Ac-GFP1 (Clontech, USA/Takara, Japan) with a 30x Glutamine linker. ZMMADSL6 was selected as a positive control in the BiFC assay as it was confirmed to interact with ZMM28 by Y2H, similar to the interaction of rice orthologs OsMADS45 with OsMADS18, respectively (18). A truncated version of ZMMADSL6 without the protein interaction domain (a leucine zipper like region in the K-domain) was generated as a negative control.

Maize seedlings were germinated and grown in Fafard Super Fine Germination Mix for 6 days in a lighted growth chamber (30 ˚C, 60% RH, 24 h light) and were transferred to a dark growth chamber (30 ˚C, 60% RH, 0 h light) and grown for an additional 4 days to V1. Seedlings were sub-irrigated with deionized water. Maize protoplasts were isolated from these seedlings and were transiently transformed by PEG-mediated transfection as described by Yoo *et al.* (19) with the addition of 0.6 M mannitol in the enzyme, WI, W5 and MMG solutions. Protoplasts were transfected with 10 pmol bait + 10 pmol prey plasmid DNA per 3 x 10^4 cells. Protoplasts were incubated on a 12-well (1 mL WI) plate for 20 h at RT before samples were analyzed.

BiFC signals were detected by flow cytometry performed using an Attune™ Flow Cytometer (Thermo Fisher Scientific, Waltham, MA, USA) with a Blue/Violet configuration (488 nm, 20 mW laser and a 405 nm, 50 mW laser). Protoplasts were first gated (R1) by forward scatter (FSC) and side scatter (SCC) to identify 10,000 intact cells (events) and subsequently analyzed for fluorescence emission measured on BL1 (530/30 nm band pass filter) and BL2 (574/26 nm band pass filter) to distinguish between cells exhibiting the BiFC signal (R2) and auto-fluorescence. At least two independent experiments were performed for each protein interaction test with the positive and negative controls present in every experiment. All experiments were designed and analyzed as single factor randomized complete blocks with $N = 4$. Significant differences were determined by analysis of variance with $P < 0.05$ comparing protein interaction partners to the truncated ZMMADSL6 (ZMMADSL6-MUT) negative control. Western blots were used to confirm expression in cells transfected with the negative control ZMMADSL6-MUT (prey) and ZMM28 (bait).

RNA-Seq of *ZmGos2-zmm28* and WT and data analysis

DP202216 was selected for in-depth molecular analysis due to its more favorable insertion region. RNA-Seq libraries were constructed from four biological replicates of WT and DP202216 youngest fully expanded leaves at V6 stage. Sequencing was performed on an Illumina HiSeq2500 (Illumina, Inc., USA) with a total read count of 154 million, and a minimum of 12 million reads per sample. RNA-Seq data were aligned to a proprietary maize B73 reference genome using Bowtie 2 (20). Overall loci abundances were estimated using the expected fragment counts metric computed by RSEM (21). Samples were vetted for quality by "Robust Principal Components based on Projection Pursuit (PP): GRID search Algorithm" in the "Scalable Robust Estimators with High Breakdown Point" R package [https://cran.r-project.org/package=rrcov]. Fold change was computed and hypothesis tests for differential expression were run using DESeq2 (22), which fits the following model:

$$K_{ij} \sim \mathrm{NB}(\mu_{ij}, \alpha_i)$$
$$\mu_{ij} = s_j q_{ij}$$
$$\log_2 q_{ij} = x_{j.} \beta_i$$

Where K_{ij} is the observed count for gene i in sample j following a Negative Binomial distribution, $(\mu_{ij}, \alpha_i, s_j, q_{ij})$ are all parameters fit to the data (see citation), $x_{j.}$ is 1 if sample j is transgenic and 0 if it is control and β_i contains the $\log_2$ fold changes for gene i across all high-nitrogen leaf samples.

All genes in the proprietary reference genome were converted to public gene model identifiers (23, 24). The DEGs were then annotated with Gene Ontology (25) terms and differential gene set enrichment was done comparing to the total publicly mapped transcript set for the entire V6 leaf data set using AgriGO (26).

Chromatin immunoprecipitation and sequencing (ChIP-seq) data analysis

Chromatin immunoprecipitation (ChIP) was performed in duplicate on the youngest fully expanded leaf from V4 WT and DP202216 maize plants using an anti-ZMM28 antibody (R743); ChIP without antibody was included as a control for each sample. Sequencing was performed on an Illumina HiSeq2500 (Illumina, Inc., USA) with a total read count of 461 million, with a minimum of 26 million reads per sample. ChIP-Seq reads were aligned to a proprietary maize B73 reference genome using Bowtie 2 (20). Alignments were then fed to MACS 2.0 (27) in order to detect differential binding in the transgenic samples. Reproducible peaks were then selected using Irreproducible Discovery Rate (IDR) analysis (28).

Direct-target analysis

Potential direct target genes (directly bound by the ZMM28 transcription factor) in V4 leaf ChIP-Seq data and select RNA-seq DEG candidates which contain CArG-boxes in the 3 kb upstream of the coding sequence were then screened based on potential function. Promoters of genes with known functional relevance and identifiable CArG sequences were synthesized (Genscript, USA) for direct target assays. Synthesized sequences were cloned into pAbAi (Clontech, USA/TAKARA, Japan) for inclusion in Yeast One-Hybrid assays with ZMM28 and Heterodimer Yeast One-Hybrid assays with ZMM28 and ZMM28 protein-protein interaction partners. Promoter sequences were integrated into the Yeast One-Hybrid Gold strain and individually transformed with a ZMM28-prey plasmid to test for protein-DNA interactions per the Yeast One-Hybrid manual. Heterodimer Yeast One-Hybrid was similarly performed, but including ZMM28 encoded on a Yeast Two-Hybrid bait plasmid (pGBK-T7) and ZMM28 protein-protein interaction partners encoded on a prey (pGAD-T7) plasmid.

Plant cell-based direct target assays were conducted in maize protoplasts. Protoplasts were isolated and transfected as described above. Reporter constructs consisted of the synthesized promoter sequences identified above transcriptionally fused to a ZsGreen1 (Clontech,

USA/TAKARA, Japan) coding sequence followed by a *pinII* terminator. Effector constructs comprised a maize *Gos2* promoter followed by a maize *ZmUbi* intron driving an effector protein coding sequence. Effector proteins were ZMM28, ZMM28 translationally fused to a 5X VP16 transcriptional activation domain, or β-glucuronidase as a negative control. Protoplasts were evaluated with flow cytometry with similar methodology to above.

Statistical analysis for physiological and biochemical experiments

For each response, separate statistical analyses were conducted using SAS software, Version 9.4 (SAS Institute Inc., Cary, NC, USA) or ASReml 3.0, R 4.0 (VSN International, Hemel Hempstead, UK, 2009). Linear mixed models were fitted per the design of each experiment, event BLUEs were estimated

Statistical assumptions of the linear mixed models (i.e. normality, independence, and homogeneous variance of the residual error) were evaluated using plots of studentized conditional residuals. For nitrate reductase, a log transformation was conducted prior to analysis to remedy departures from assumptions. Results for nitrate reductase were back-transformed to the original data scale prior to reporting. The assumptions were satisfied for all other responses. Each of the transgenic events was compared to the respective WT control using two-tailed t-tests of differences between the estimates. In experiments with multiple hybrids, when interaction of event and hybrid was found significant, the comparisons were conducted within each hybrid. The approximated degrees of freedom for the statistical tests were derived using the Kenward-Roger method (29).

Supplementary Results

A

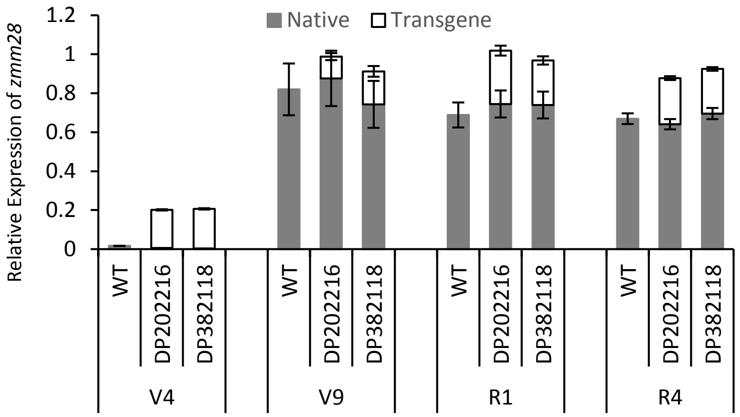

B

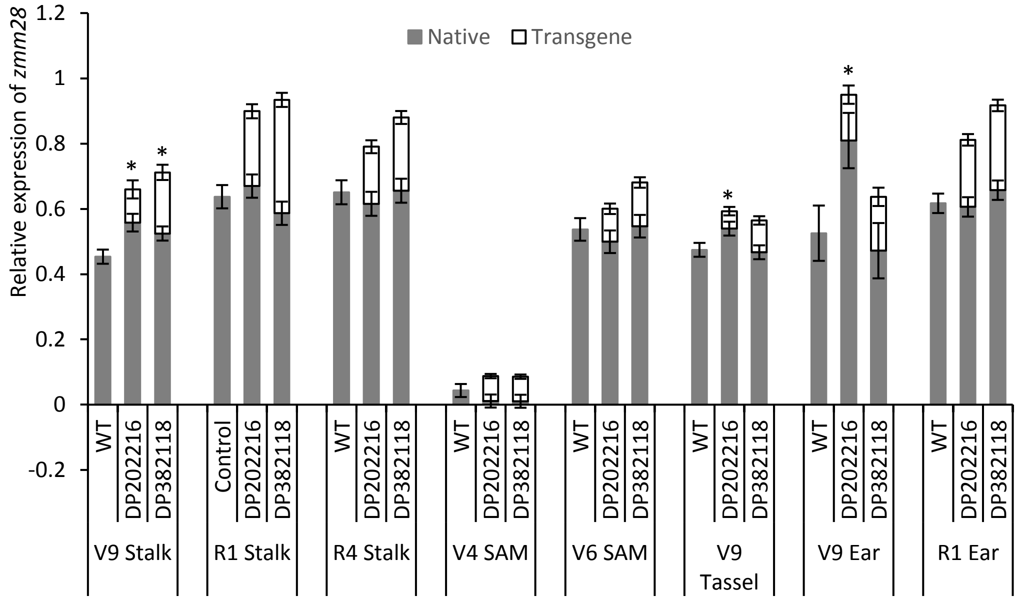

C

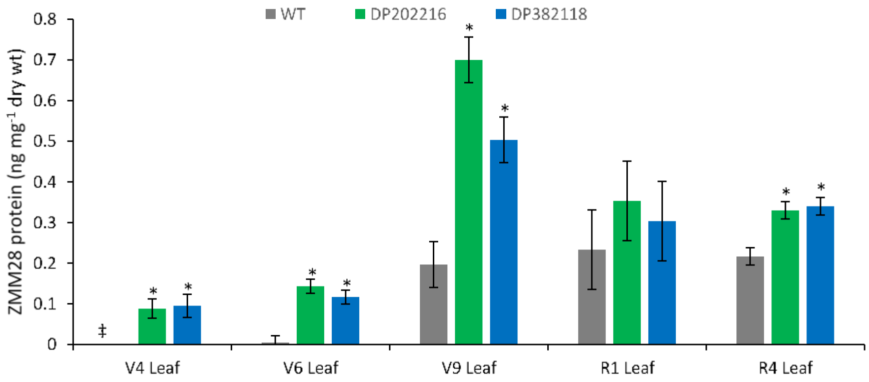

D

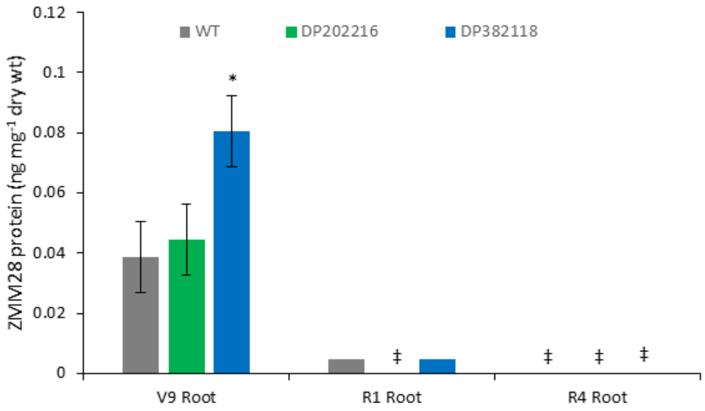

E

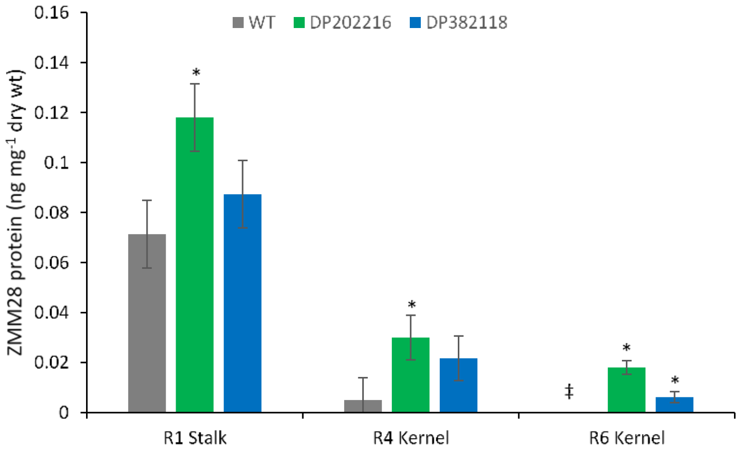

Fig. S1. Levels of *zmm28* mRNA and protein (ng mg^{-1} dry weight) in field-plot grown plants: (A) relative WT and transgenic mRNA expression in root tissue; (B) relative mRNA expression in stalk, shoot apical meristem (SAM), tassel and ear tissues; (C) ZMM28 protein concentration in leaf; (D) root; (E) stalk and kernel tissues. WT, DP202216, and DP382118 in (C - E). Error bars represent standard error. $N = 3$; $*P < 0.05$, and ‡ = 0.

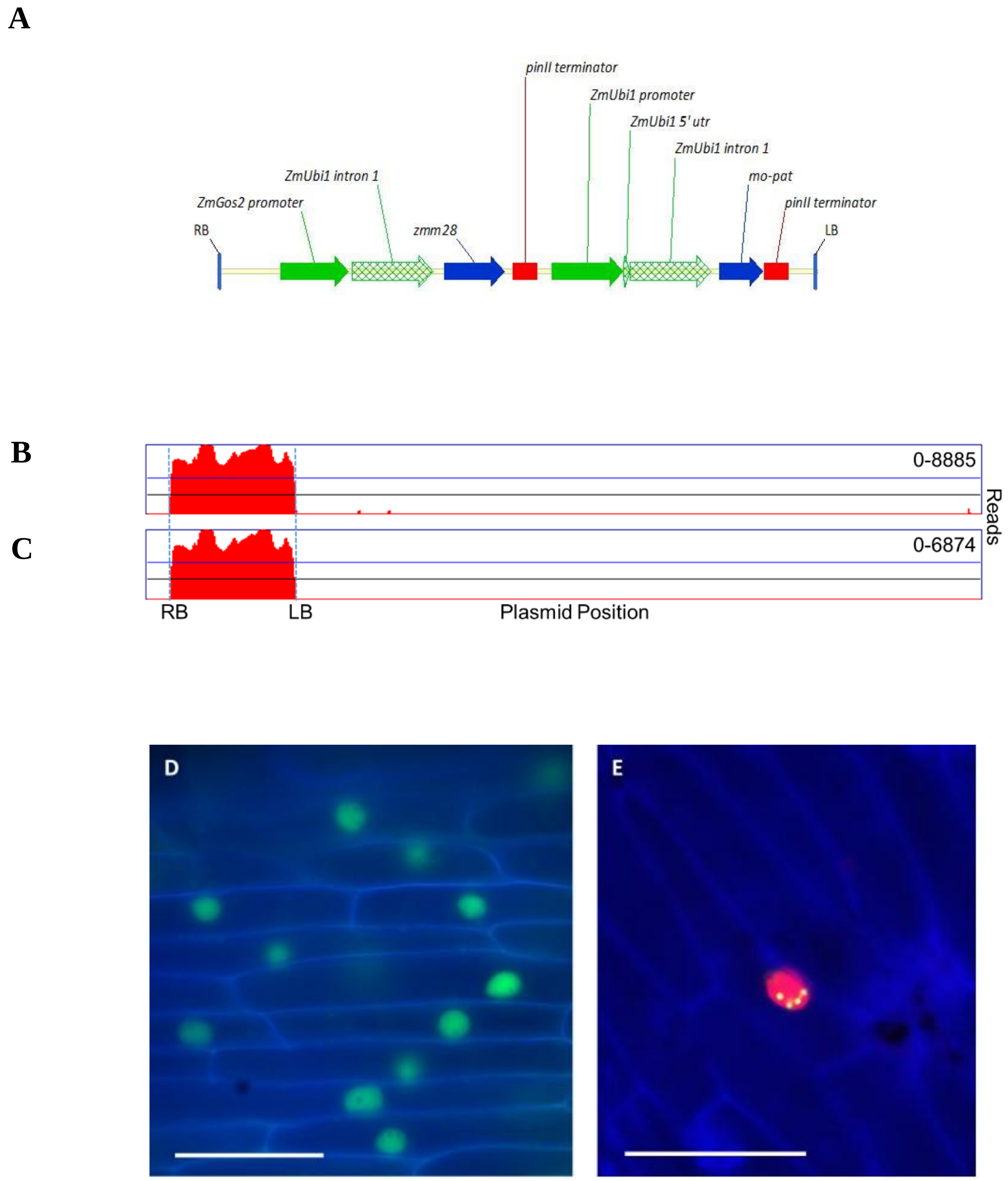

Fig. S2. (A) Linear map of *ZmGos2-zmm28* trait gene cassette T-DNA from right border (RB) to left border (LB). Representative sequence coverage graphs of *ZmGos2-zmm28* events (B) DP202216 and (C) DP382118. Base coverage is shown in red with the minimum and maximum of coverage represented by a number range in the upper right of each coverage panel. Black and blue horizontal lines represent 10 x and 100 x sequence coverage, respectively, at each base along the x-axis representing the transformation plasmid. The RB and LB delineate the T-DNA region. Fluorescent micrograph of stably transformed leaf cells (D) and a transiently transformed maize coleoptile cell (E). Expression of *ZmGos2-zmm28*:AcGFP1 (green) shows localization of ZMM28 within the nucleus (D and E). Red fluorescence (E) denotes localization of the maize histone H2B with a C-terminal mKate2 fusion in the transiently transformed maize coleoptile cell. The cell wall outline is visible in blue using a DAPI filter set highlighting cell wall autofluorescence. The white scale bars represent 50 µm.

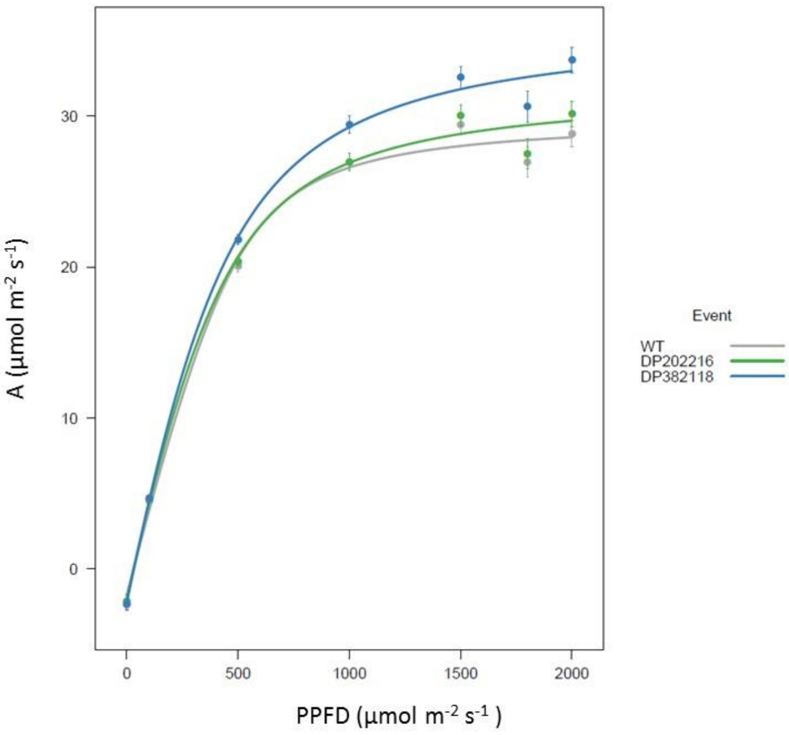

Fig. S3. Photosynthesis rate (A) vs. light (PPFD) response curve of WT, DP202216 and DP382118 maize plants measured from plants grown in field-pots at R4 stage. Error bars represent the standard error, $N = 60$.

Table S1. Summary of *ZmGos2-zmm28* vs. WT hybrid field trials from 2014 – 2017

Year	Number of germplasm backgrounds	Number of locations	Number of replicates	Total data points $event^{-1}$
2014	6	10	3	180
2015	12	15	3	414
2016	18	14	2-3	290
2017	12	19	2	356
Total	48	58		1240

Table S2. The percent change in secondary (breeder) traits of *ZmGos2-zmm28* hybrid plants (DP202216 and DP382118) vs. WT

	**DP202216**	**DP382118**
**Secondary Trait**	**% of Wild Type**	
Plant Height	100.7*	100.7*
Heat Units to Shed	100.8*	100.4*
Heat Units to Silk	100.7*	100.4*
Root Lodging	95.0	101.0
Grain Moisture	101.3*	101.5*

Data were collected from field yield trials. *N* = 1719 for plant height, *N* = 389 for heat units to shed, *N* = 351 for heat units to silk, *N* = 44 for root lodging and *N* = 2381 for grain moisture. *$P < 0.05$.

Table S3. Maize protoplast Bimolecular Fluorescence Complementation assay summary

GOI ID	Protein description	Negative control cell count	Positive control cell count (Positive vs. negative p value)	GOI cell count (GOI vs. negative p value)	GOI vs. positive *P*-value)	Summary
Zm00001d041781	ZmZAG2	65	155 (0.027)	125 (0.053)	0.195	-
Zm00001d017614	ZmMADS6	375	1589 (0.002)	1899 (0.002)	0.146	+
Zm00001d018667	ZmZAPL	491	1538 (0.037)	2161 (0.010)	0.285	+
Zm00001d022088	ZMM28	483	878 (0.010)	1014 (0.034)	0.429	+
Zm00001d028217	ZmM5	2085	4728 (0.007)	4512 (0.004)	0.675	+
Zm00001d031620	ZmMADSL6	2396	N/A (N/A)	4059 (0.002)	N/A	+
Zm00001d021057	ZmMADS7-LIKE	421	1321 (0.006)	438 (0.427)	0.010	-
Zm00001d034047	ZmMADS24	586	2024 (0.044)	2099 (0.035)	0.764	+
Zm00001d044899	ZmMADS47-LIKE	483	878 (0.010)	254 (0.047)	0.006	-
Zm00001d027957	ZmM47	483	878 (0.010)	1085 (0.005)	0.107	+
Zm00001d037925	ZmSF2	483	878 (0.010)	715 (0.077)	0.363	-
Zm00001d022164	ZmSFT-LIKE	421	1321 (0.006)	2328 (0.016)	0.016	+

Values represent BiFC positive cell counts from selected gate strategies out of 10,000 cells in representative flow cytometry experiments. Negative- and positive-control cell counts represent fluorescent cells in protoplast populations co-transfected with BiFC fusion constructs of truncated ZmMADSL6 and ZMM28 or full length ZmMADSL6 and ZMM28, respectively. GOI = gene of interest. +/- indicates whether the BiFC assay was concluded to be positive (+) or negative (-) based on the p-value calculations.

Table S4. List of differentially expressed genes at a 95% confidence interval in DP202216 vs control V6 leaf tissue

ID	Base mean	log_2 fold change	adjusted p-value
Zm00001d022088	366.7	2.161	0
Zm00001d023455	399.2	1.309	0
Zm00001d023456	775.4	1.175	0
Zm00001d038273	268.1	1.056	0
Zm00001d004053	788.2	1.054	0
Zm00001d029183	467.1	0.885	0.018
Zm00001d051194	422.1	0.735	0.006
Zm00001d033132	1007.7	0.719	0
Zm00001d029215	401.8	0.71	0.049
Zm00001d033543	949.1	0.71	0.01
Zm00001d053925	887.4	0.695	0.003
Zm00001d028269	226.6	0.651	0
Zm00001d033544	255.6	0.643	0
Zm00001d034015	844.9	0.617	0
Zm00001d027743	384.8	0.601	0
Zm00001d048311	698.4	0.565	0
Zm00001d053787	4263	0.508	0.019
Zm00001d047256	331.2	0.49	0
Zm00001d048720	258.8	0.44	0.017
Zm00001d023426	2247.4	0.436	0.004
Zm00001d004331	771.7	0.43	0.049
Zm00001d050748	566.4	0.424	0
Zm00001d010321	1082.7	0.416	0.008
Zm00001d004894	15128.1	0.403	0
Zm00001d042346	506.7	0.401	0.045
Zm00001d031657	1064.8	0.393	0.002
Zm00001d008178	262	0.377	0.042
Zm00001d043044	3741.8	0.371	0.001
Zm00001d018623	374.5	0.37	0.002
Zm00001d043095	820.2	0.369	0
Zm00001d029062	448.4	0.354	0.018
Zm00001d011900	1851.3	0.347	0.001
Zm00001d010672	3578.8	0.333	0.003
Zm00001d011183	6346	0.324	0.049
Zm00001d037103	1178.1	0.322	0.039
Zm00001d009028	28412.8	0.318	0.038
Zm00001d013937	3658	0.318	0.008

Zm00001d002873	412.1	0.315	0.01
Zm00001d037362	731.7	0.311	0.039
Zm00001d026404	8690.7	0.31	0.028
Zm00001d047255	2253.8	0.309	0.024
Zm00001d031253	5805.9	0.309	0
Zm00001d027576	856.8	0.306	0.011
Zm00001d052595	12058.5	0.304	0.002
Zm00001d013367	1046.5	0.301	0.007
Zm00001d046001	2845.8	0.297	0.049
Zm00001d008963	418.8	0.296	0.049
Zm00001d048515	366	0.29	0.043
Zm00001d018779	5833.9	0.289	0.028
Zm00001d052595	5155.7	0.289	0.02
Zm00001d042049	5095.7	0.288	0.016
Zm00001d040242	573.1	0.287	0.022
Zm00001d042697	29924.1	0.285	0
Zm00001d019518	2389.6	0.283	0.004
Zm00001d048313	568.8	0.283	0.034
Zm00001d033150	2844.8	0.281	0
Zm00001d007858	4471.7	0.28	0
Zm00001d003588	1202.7	0.28	0.005
Zm00001d036903	1562.2	0.279	0.019
Zm00001d031484	3091.2	0.276	0
Zm00001d018157	2066.8	0.275	0.012
Zm00001d005814	3964.9	0.275	0
Zm00001d033338	1889.2	0.275	0.028
Zm00001d053446	1126.3	0.272	0.027
Zm00001d026603	26238.1	0.271	0
Zm00001d027841	19250.6	0.27	0.016
Zm00001d030048	4061	0.269	0.001
Zm00001d005346	4426.3	0.267	0.025
Zm00001d023706	4737.6	0.267	0
Zm00001d042050	13900.2	0.266	0.013
Zm00001d042533	870.8	0.262	0.019
Zm00001d022590	756.8	0.261	0.024
Zm00001d045431	3088.4	0.26	0.045
Zm00001d047743	2167.9	0.26	0
Zm00001d036738	4088.9	0.251	0
Zm00001d018274	2320	0.251	0.02
Zm00001d035003	9178.4	0.251	0.028
Zm00001d027321	906.8	0.25	0.013
Zm00001d014284	2863.7	0.249	0
Zm00001d036340	3635.8	0.248	0.039

Zm00001d003767	684.3	0.248	0.021
Zm00001d031997	2770.7	0.246	0.022
Zm00001d019479	7547.5	0.245	0
Zm00001d024148	10915.4	0.244	0.013
Zm00001d038579	22011	0.243	0
Zm00001d037273	2559.2	0.24	0.028
Zm00001d025845	682.1	0.239	0.033
Zm00001d027422	1300.4	0.239	0.049
Zm00001d024519	15382.2	0.238	0
Zm00001d040163	4199.3	0.238	0
Zm00001d012868	2451.1	0.237	0.001
Zm00001d021763	46089.8	0.235	0.049
Zm00001d035761	2876.1	0.232	0.028
Zm00001d032301	1829.9	0.231	0.003
Zm00001d028562	7597.8	0.231	0.048
Zm00001d034538	1219.9	0.231	0.042
Zm00001d048116	1310.7	0.23	0.003
Zm00001d048998	33243	0.226	0.003
Zm00001d049490	896.9	0.225	0.049
Zm00001d015975	1389.6	0.224	0.03
Zm00001d021435	101585.8	0.223	0.001
Zm00001d015613	1517.4	0.221	0.049
Zm00001d039258	45911.3	0.221	0.023
Zm00001d007267	22445.6	0.22	0
Zm00001d033383	8820.9	0.22	0.04
Zm00001d026645	774.2	0.22	0.028
Zm00001d023757	3788	0.219	0.026
Zm00001d034005	1328	0.218	0.017
Zm00001d022381	1437.7	0.215	0.028
Zm00001d031962	1481.3	0.214	0.012
Zm00001d005446	17349.9	0.214	0.017
Zm00001d027511	1065.8	0.211	0.034
Zm00001d017178	1469.3	0.21	0.02
Zm00001d038947	2902.2	0.209	0.007
Zm00001d009982	2640.8	0.209	0.006
Zm00001d034739	1282.4	0.208	0.033
Zm00001d044745	2078.9	0.208	0.002
Zm00001d038491	1871.3	0.208	0.007
Zm00001d014445	2012.3	0.207	0.029
Zm00001d039745	2541.4	0.207	0.049
Zm00001d038485	2954.2	0.207	0.02
Zm00001d012287	3196.9	0.205	0.042
Zm00001d027694	4079	0.204	0.002

Zm00001d003470	2149.7	0.204	0.049
Zm00001d019180	1691.1	0.203	0.001
Zm00001d007394	2809.7	0.203	0.046
Zm00001d053432	14717.4	0.202	0
Zm00001d039900	3507.8	0.2	0.004
Zm00001d050810	2267.9	0.2	0.013
Zm00001d016802	3694.7	0.2	0.024
Zm00001d004978	1355.2	0.199	0.049
Zm00001d028924	2314.7	0.199	0.017
Zm00001d002815	6335.2	0.198	0.034
Zm00001d029065	1335.9	0.198	0.033
Zm00001d052184	1429.5	0.197	0.005
Zm00001d038337	1709	0.193	0.049
Zm00001d003713	843.8	0.193	0.049
Zm00001d031953	2062.3	0.193	0.049
Zm00001d053576	1070.5	0.191	0.036
Zm00001d000123	4446.2	0.191	0.01
Zm00001d045431	12598.3	0.19	0.039
Zm00001d036630	26955.9	0.187	0
Zm00001d048515	4138.4	0.186	0.044
Zm00001d021310	10622.2	0.185	0.009
Zm00001d038037	13570.3	0.184	0.005
Zm00001d042353	1578.4	0.183	0.029
Zm00001d050150	1799.2	0.183	0.014
Zm00001d025545	10158.4	0.182	0
Zm00001d012168	1918.6	0.182	0.006
Zm00001d011581	2421.3	0.176	0.013
Zm00001d018030	12550.6	0.173	0.012
Zm00001d018145	15856.7	0.171	0.012
Zm00001d040221	4822.7	0.17	0.001
Zm00001d033594	4970	0.17	0.006
Zm00001d008625	3404.2	0.17	0.015
Zm00001d032380	6439.8	0.169	0.042
Zm00001d045575	9388.9	0.165	0.012
Zm00001d042526	12885.2	0.164	0.024
Zm00001d043168	9130.5	0.164	0.014
Zm00001d053545	13321.4	0.162	0.012
Zm00001d053981	2247.8	0.162	0.031
Zm00001d017746	3272.3	0.162	0.023
Zm00001d012083	3531.8	0.161	0.002
Zm00001d024718	1276.1	0.16	0.039
Zm00001d021621	29252	0.16	0.033
Zm00001d015004	3642	0.159	0.031

Zm00001d039131	6755.2	0.158	0.013
Zm00001d044970	11313.1	0.158	0.015
Zm00001d018401	3854.9	0.156	0.007
Zm00001d015975	2687.4	0.15	0.024
Zm00001d013428	6916.2	0.15	0.002
Zm00001d021246	8907.5	0.148	0.041
Zm00001d007921	1700.3	0.146	0.045
Zm00001d027511	11194	0.144	0.039
Zm00001d027309	2776.5	0.14	0.031
Zm00001d025544	4067.6	0.138	0.04
Zm00001d039276	2421.5	0.137	0.049
Zm00001d003512	7940.7	0.134	0.016
Zm00001d053861	4328.4	0.13	0.048
Zm00001d038894	18625	0.129	0.007
Zm00001d051321	5842.9	0.128	0.039
Zm00001d016826	6846.9	0.126	0.031
Zm00001d016854	3875.9	0.122	0.032
Zm00001d017435	4798.8	0.121	0.018
Zm00001d048373	16010.1	0.109	0.019
Zm00001d018901	18599.9	0.104	0.043
Zm00001d019454	18340.3	0.1	0.046
Zm00001d031899	38207.2	0.097	0.036
Zm00001d045706	13660.8	-0.114	0.003
Zm00001d035869	3703.4	-0.169	0.035
Zm00001d053262	2715	-0.177	0.034
Zm00001d017958	1986.1	-0.179	0.042
Zm00001d007113	2081.6	-0.182	0.007
Zm00001d051650	1133.1	-0.205	0.025
Zm00001d015138	60518.8	-0.222	0
Zm00001d030103	1549.1	-0.225	0.034
Zm00001d021846	1819.7	-0.226	0
Zm00001d041576	849.3	-0.252	0.039
Zm00001d005391	618.2	-0.255	0.019
Zm00001d009022	4240	-0.264	0.023
Zm00001d041343	1309.4	-0.268	0.045
Zm00001d039965	528.6	-0.274	0.025
Zm00001d029047	509.7	-0.275	0.021
Zm00001d028230	2507.9	-0.284	0.012
Zm00001d013243	725.6	-0.286	0.04
Zm00001d032926	458.9	-0.299	0.034
Zm00001d045667	1861.6	-0.302	0
Zm00001d021846	701.7	-0.303	0.025
Zm00001d033469	934.7	-0.312	0.032

Zm00001d019563	3360	-0.314	0.007
Zm00001d003866	945.1	-0.316	0.008
Zm00001d039325	1802.7	-0.321	0
Zm00001d023516	69804.9	-0.324	0.036
Zm00001d050918	301	-0.325	0.029
Zm00001d025253	331.5	-0.346	0.04
Zm00001d024499	1459.3	-0.364	0.029
Zm00001d045948	478.6	-0.374	0.013
Zm00001d021569	473.1	-0.376	0.024
Zm00001d053327	601.3	-0.377	0.002
Zm00001d047208	736.1	-0.379	0
Zm00001d007687	1551.8	-0.385	0.001
Zm00001d015126	1333.7	-0.395	0.005
Zm00001d034781	288.2	-0.403	0.006
Zm00001d016655	1486.6	-0.412	0.049
Zm00001d043517	322.3	-0.426	0.013
Zm00001d045667	288.4	-0.427	0.049

Base Mean = the mean of counts of all samples, normalized for sequencing depth.
30 transcripts are not represented because they either map to the same gene or do not map to the B73 reference genome.

References

1. de Pater BS, *et al.* (1992) The promoter of the rice gene GOS2 is active in various different monocot tissues and binds rice nuclear factor ASF-1. *Plant J* 2(6):837-844.
2. Christensen AH, Sharrock RA, & Quail PH (1992) Maize polyubiquitin genes: structure, thermal perturbation of expression and transcript splicing, and promoter activity following transfer to protoplasts by electroporation. *Plant Mol Biol* 18(4):675-689.
3. An G, *et al.* (1989) Functional analysis of the 3'control region of the potato wound-inducible proteinase inhibitor II gene. *Plant Cell* 1(1):115-122.
4. Zastrow-Hayes GM, *et al.* (2015) Southern-by-Sequencing: A Robust Screening Approach for Molecular Characterization of Genetically Modified Crops. *The Plant Genome* 8(1):0.
5. Cullis B, *et al.* (1998) Spatial Analysis of Multi-Environment Early Generation Variety Trials. *Biometrics* 54(1):1-18.
6. Gilmour A, *et al.* (2009) *ASReml user guide release 3.0* (VSN Int Ltd, Hemel Hempstead, UK).
7. Gilmour AR, Thompson R, & Cullis BR (1995) Average information REML: an efficient algorithm for variance parameter estimation in linear mixed models. *Biometrics* 51(4):1440-1450.
8. Sun J, Gibson KM, Kiirats O, Okita TW, & Edwards GE (2002) Interactions of nitrate and CO2 enrichment on growth, carbohydrates, and rubisco in Arabidopsis starch mutants. Significance of starch and hexose. *Plant Physiol* 130(3):1573-1583.
9. Hoagland DR & Arnon DI (1950) The water-culture method for growing plants without soil. *Circular. California Agricultural Experiment Station* 347(2nd edit).
10. Shcherbo D, *et al.* (2009) Far-red fluorescent tags for protein imaging in living tissues. *Biochem J* 418(3):567-574.
11. Lawit SJ, Wych HM, Xu D, Kundu S, & Tomes DT (2010) Maize DELLA proteins dwarf plant8 and dwarf plant9 as modulators of plant development. *Plant Cell Physiol* 51(11):1854-1868.
12. Lawit SJ, Chamberlin MA, Agee A, Caswell ES, & Albertsen MC (2013) Transgenic manipulation of plant embryo sacs tracked through cell-type-specific fluorescent markers: cell labeling, cell ablation, and adventitious embryos. *Plant reproduction* 26(2):125-137.
13. Miranda KM, Espey MG, & Wink DA (2001) A rapid, simple spectrophotometric method for simultaneous detection of nitrate and nitrite. *Nitric Oxide* 5(1):62-71.
14. Sun J, *et al.* (2014) Inconsistency of mesophyll conductance estimate causes the inconsistency for the estimates of maximum rate of Rubisco carboxylation among the linear, rectangular and non-rectangular hyperbola biochemical models of leaf photosynthesis--a case study of CO(2) enrichment and leaf aging effects in soybean. *Plant Sci* 226:49-60.
15. Maxwell K & Johnson GN (2000) Chlorophyll fluorescence—a practical guide. *Journal of Experimental Botany* 51(345):659-668.
16. Kanai R & Edwards GE (1973) Separation of mesophyll protoplasts and bundle sheath cells from maize leaves for photosynthetic studies. *Plant Physiol* 51(6):1133-1137.
17. Krall JP, Edwards GE, & Andreo CS (1989) Protection of Pyruvate,Pi Dikinase from Maize against Cold Lability by Compatible Solutes. *Plant Physiol* 89(1):280-285.
18. Fornara F, *et al.* (2004) Functional characterization of OsMADS18, a member of the AP1/SQUA subfamily of MADS box genes. *Plant Physiol* 135(4):2207-2219.

19. Yoo SD, Cho YH, & Sheen J (2007) Arabidopsis mesophyll protoplasts: a versatile cell system for transient gene expression analysis. *Nat Protoc* 2(7):1565-1572.
20. Langmead B, Trapnell C, Pop M, & Salzberg SL (2009) Ultrafast and memory-efficient alignment of short DNA sequences to the human genome. *Genome Biol* 10(3):R25.
21. Li B & Dewey CN (2011) RSEM: accurate transcript quantification from RNA-Seq data with or without a reference genome. *BMC Bioinformatics* 12(1):323.
22. Love MI, Huber W, & Anders S (2014) Moderated estimation of fold change and dispersion for RNA-seq data with DESeq2. *Genome Biol* 15(12):550.
23. Andorf CM*, et al.* (2016) MaizeGDB update: new tools, data and interface for the maize model organism database. *Nucleic Acids Res* 44(D1):D1195-1201.
24. Jiao Y*, et al.* (2017) Improved maize reference genome with single-molecule technologies. *Nature* 546:524.
25. Ashburner M*, et al.* (2000) Gene ontology: tool for the unification of biology. The Gene Ontology Consortium. *Nat Genet* 25(1):25-29.
26. Du Z, Zhou X, Ling Y, Zhang Z, & Su Z (2010) agriGO: a GO analysis toolkit for the agricultural community. *Nucleic Acids Res* 38(Web Server issue):W64-70.
27. Zhang Y*, et al.* (2008) Model-based analysis of ChIP-Seq (MACS). *Genome Biol* 9(9):R137.
28. Li Q, Brown JB, Huang H, & Bickel PJ (2011) Measuring reproducibility of high-throughput experiments. *The Annals of Applied Statistics*:1752-1779.
29. Kenward MG & Roger JH (2009) An improved approximation to the precision of fixed effects from restricted maximum likelihood. *Computational Statistics & Data Analysis* 53(7):2583-2595.
